# Membrane Binding by CHMP7 Coordinates ESCRT-III-Dependent Nuclear Envelope Reformation

**DOI:** 10.1016/j.cub.2016.07.039

**Published:** 2016-10-10

**Authors:** Yolanda Olmos, Anna Perdrix-Rosell, Jeremy G. Carlton

**Affiliations:** 1 Division of Cancer Studies, King’s College London, London SE1 1UL, UK

**Keywords:** cell biology, mitosis, ESCRT-III, nuclear envelope, cell division

## Abstract

In addition to its role in membrane abscission during cytokinesis, viral budding, endosomal sorting, and plasma membrane repair [1], the endosomal sorting complex required for transport-III (ESCRT-III) machinery has recently been shown to seal holes in the reforming nuclear envelope (NE) during mitotic exit [2, 3]. ESCRT-III also acts during interphase to repair the NE upon migration-induced rupture [4, 5], highlighting its key role as an orchestrator of membrane integrity at this organelle. While NE localization of ESCRT-III is dependent upon the ESCRT-III component CHMP7 [3], it is unclear how this complex is able to engage nuclear membranes. Here we show that the N terminus of CHMP7 acts as a novel membrane-binding module. This membrane-binding ability allows CHMP7 to bind to the ER, an organelle continuous with the NE, and it provides a platform to direct NE recruitment of ESCRT-III during mitotic exit. CHMP7’s N terminus comprises tandem Winged-Helix domains [6], and, by using homology modeling and structure-function analysis, we identify point mutations that disrupt membrane binding and prevent both ER localization of CHMP7 and its subsequent enrichment at the reforming NE. These mutations also prevent assembly of downstream ESCRT-III components at the reforming NE and proper establishment of post-mitotic nucleo-cytoplasmic compartmentalization. These data identify a novel membrane-binding activity within an ESCRT-III subunit that is essential for post-mitotic nuclear regeneration.

## Highlights

• CHMP7’s N terminus is a membrane-binding domain
• Membrane binding allows CHMP7 to localize to the ER
• ER binding is necessary for CHMP7 localization to the reforming NE
• CHMP7’s membrane binding is necessary for regeneration of the post-mitotic NE

## Graphical Abstract

**Figure:**
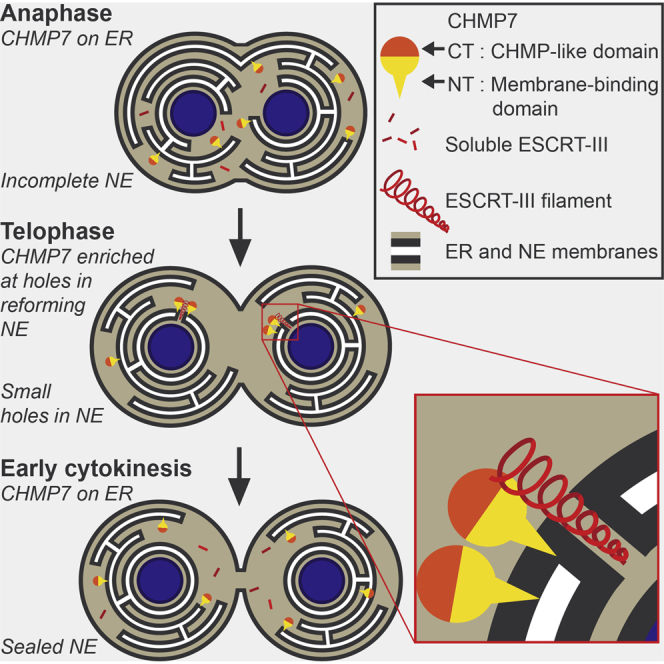

## Teaser

ESCRT-III is necessary for closing small holes in the reforming nuclear envelope during mitotic exit. Olmos et al. show that CHMP7, a key component of this machinery, contains a novel membrane-binding domain that is necessary for the assembly of ESCRT-III at this organelle and for the regeneration of a sealed nuclear envelope during mitotic exit.

## Results and Discussion

**Figure 1:**
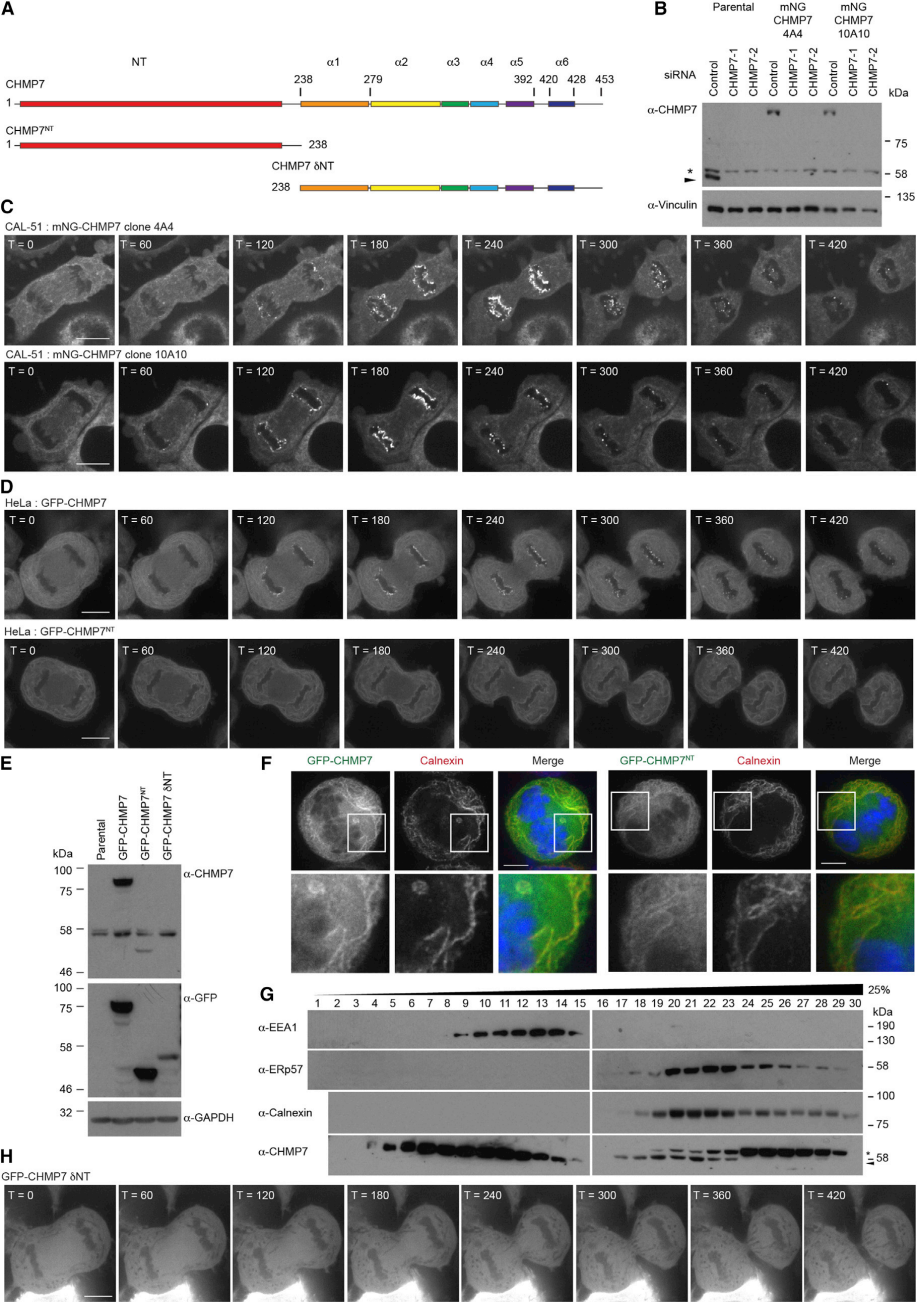
CHMP7 Is an ER-Localized Protein that Is Enriched at the NE during Mitotic Exit (A) Schematic depicting constructs used in this study. (B and C) CAL-51 cells edited to express mNG-CHMP7 were resolved and examined by western blotting with anti-CHMP7 and anti-Vinculin (B) or imaged live (C). In this and all other figures, endogenous CHMP7 is marked by an arrowhead (^∗^, non-specific band). (D–F) HeLa cells stably expressing GFP-CHMP7 and GFP-CHMP7^NT^ were imaged live (D), lysed, resolved, and examined by western blotting with anti-GFP, anti-CHMP7, or anti-GAPDH antisera (E) or fixed and stained with anti-Calnexin and DAPI (F). Images in (D) are representative of all cells imaged and 22/22 (GFP-CHMP7) and 21/21 (GFP-CHMP7^NT^) captured movies. Co-localization of GFP-CHMP7 and GFP-CHMP7^NT^ with Calnexin was observed in 7/7 and 13/13 scored cells, respectively. (G) Post-nuclear supernatants from Cos7 cells were fractionated through a continuous iodixanol gradient and analyzed by western blotting with the indicated antisera. (H) HeLa cells stably expressing GFP-CHMP7 δNT were imaged live. Images are representative of all cells imaged and 5/5 captured movies. Time interval is presented in seconds post-cortical ingression. In all micrographs, the scale bar represents 10 μm. See also Figure S1.

CHMP7 is unique among endosomal sorting complex required for transport-III (ESCRT-III) subunits in that it contains an extended N terminus (NT) (Figures 1A and S1A) that we hypothesized may be important during its role in nuclear envelope (NE) regeneration. Using a dual-nickase CRISPR/Cas9 approach [7], we edited the CHMP7 locus in CAL-51 cells to produce a homozygous N-terminal fusion of monomeric-NeonGreen (mNG) [8] to CHMP7 under the control of its endogenous promoter (Figures 1B and S1B–S1I). These cells grew normally, suggesting that N-terminal tagging of CHMP7 is benign. We imaged living mNG-CHMP7 cells and found that, while CHMP7 was recruited to the NE during mitotic exit, in addition to a cytoplasmic pool, it decorated ER membranes in interphase and mitotic cells (Figures 1C and S1J; Movie S1). Stably expressed GFP-CHMP7 localized similarly (Figures 1D–1F and S1K–S1M). We saw no localization of GFP-CHMP7 to the midbody (Figure S1N). CHMP7-antisera failed to detect small interfering RNA (siRNA)-sensitive immunofluorescence signal; however, we could detect endogenous CHMP7 in ER fractions from homogenized cells (Figures 1G and S1O).

*S. cerevisiae* Chm7 was recently shown to localize to the ER [6], suggesting that this localization is evolutionarily conserved. During NE reformation, all other ESCRT-III subunits are recruited from the cytoplasm [2, 3]; given that the NE is formed from the ER [9, 10], a pre-existing ER localization for CHMP7 suggested a platform from which this recruitment could occur. Analysis of HeLa cells stably expressing GFP-CHMP7^NT^ or mCh-CHMP7^NT^ revealed that CHMP7’s N terminus directed localization to the ER, but this truncated protein exhibited little stabilization at the reforming NE (Figures 1D and S2A–S2D; Movie S2). In contrast, the C terminus of CHMP7 (GFP-CHMP7 δNT) was cytosolic and displayed neither ER localization nor stabilization at the reforming NE (Figure 1H; Movie S2), despite containing the CHMP4B/ESCRT-III interaction domain [11]. CHMP7 is responsible for recruiting downstream ESCRT-III components to the reforming NE through CHMP4B. Fusion of siRNA-resistant CHMP7^NT^ to CHMP4B directed cytoplasmic CHMP4B to the mitotic ER and restored its enrichment at sites of annular fusion at the forming NE, in the absence of endogenous CHMP7 (Figures S2E–S2G).

Analysis of the secondary structure of CHMP7^NT^ has revealed the presence of tandem winged helix (WH) domains [6, 12], resembling those found in ESCRT-II subunits (Figure S3A). During endosomal sorting, membrane-anchored ESCRT-II serves to recruit ESCRT-III to endosomes through interaction of the second WH domain of VPS25 with the ESCRT-III component VPS20/CHMP6 [13, 14]. As CHMP7 initiates ESCRT-III assembly at the NE, we wondered whether its N terminus acted as a membrane adaptor at this organelle. HHpred (https://toolkit.tuebingen.mpg.de/hhpred) alignments of CHMP7 matched its NT to VPS25 [6], and, by aligning predicted secondary structural elements in CHMP7 to those present in the crystal structure of VPS25, we noted an evolutionarily conserved extension of the loop between the β2-β3 hairpin in the first WH domain of CHMP7^NT^ (Figure S3A).

**Figure 2:**
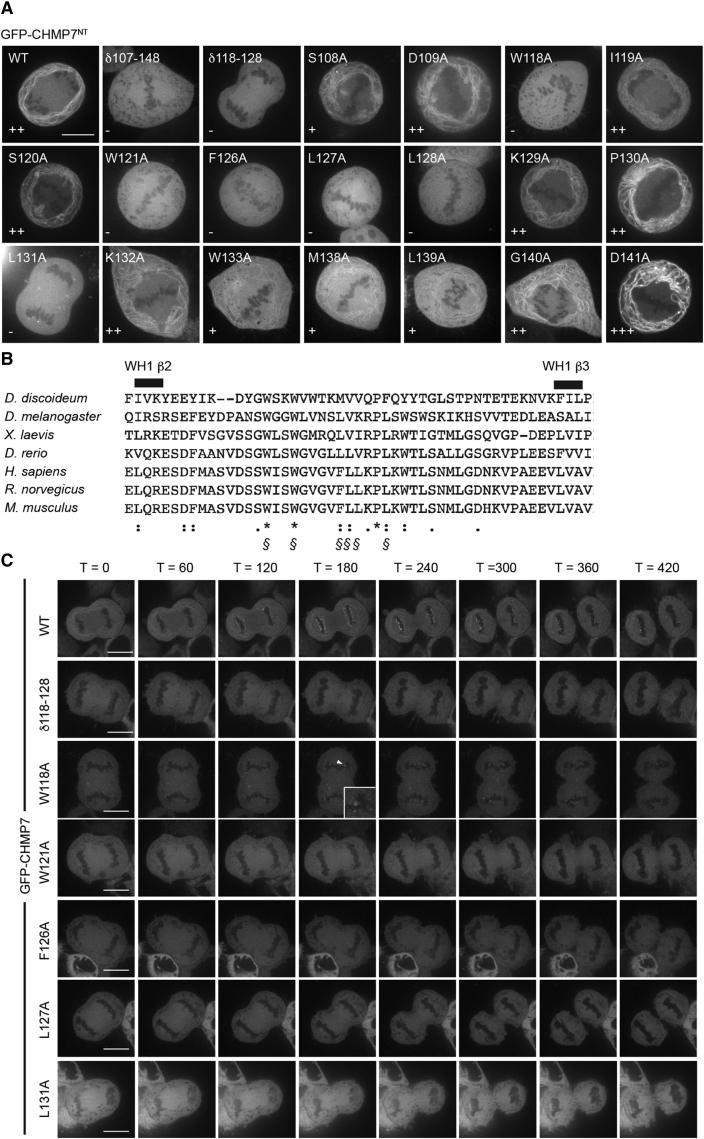
Mapping Activities in CHMP7^NT^ that Govern ER Localization (A) HeLa cells were transfected with the indicated GFP-CHMP7^NT^ plasmids and imaged live; individual residues within the β2-β3 insertion that disrupted ER localization when previously mutated in blocks of four (Figures S3B–S3D) were mutated to alanine. Reticular localization is indicated as follows: −, none/cytoplasmic; +, reduced; ++, normal; and +++, enhanced). Reticular localization was observed in the indicated number of captured images from three independent experiments. NT, 11/11; δ107–148, 0/11; δ 118–128, 0/13; S108A, 12/12; D109A, 11/11; W118A, 0/13; I119A, 11/11; S120A, 11/11; W121A, 0/12; F126A, 0/12; L127A, 0/14; L128A, 0/14; K129A, 13/13; P130A, 13/13; L131A, 0/12; K132A, 12/13; W133A, 13/14; M138A, 15/15; L139A, 11/12; G140A, 16/16; D141A, 13/13. (B) Sequence alignment of the insertion between β2 and β3 in the CHMP7^NT^ WH1 domain from the indicated organisms. Conservation extent is as follows: ^∗^, complete; :, strongly similar; ⋅, weakly similar; §, residues necessary for ER localization. (C) HeLa cells expressing the indicated GFP-CHMP7 constructs were imaged live (time in seconds post-cortical ingression). Images are representative of 3/3 acquired movies and 50/50 scored cells per mutation. Limited enrichment on the telophase NE (boxed) was observed for GFP-CHMP7 W118A, suggesting that some degree of ER localization persists in this case. In all micrographs, the scale bar represents 10 μm. See also Figures S2 and S3.

Deletions through CHMP7^NT^ were poorly expressed (Figures S2C and S2D), so we performed scanning mutagenesis through CHMP7^NT^ to identify ER localization determinants (Figures S3B and S3C). We discovered 12 mutagenic tetrads that prevented ER localization, five of which were found on the extended loop in CHMP7^NT^-WH1. We created a homology model of CHMP7^NT^ (lacking the extended loop) and mapped the remaining mutations to regions that were either in or engaged with residues on the WH1 β2-β3 hairpin (Figure S3D). Deletion of this loop (CHMP7 δ107–148) prevented ER localization (Figure 2A). In case alanine changes in blocks of four prevented proper folding, we mutated individual residues within this loop to fine-map determinants of ER localization. Mutation of six evolutionarily conserved hydrophobic residues (W118, W121, F126, L127, L128, and L131) or deletion of this hydrophobic stretch (δ118–128) prevented ER localization of CHMP7^NT^ (Figures 2A and 2B). These mutations prevented full-length CHMP7 from localizing to the ER and becoming enriched at the reforming NE (Figure 2C; Movie S3).

**Figure 3:**
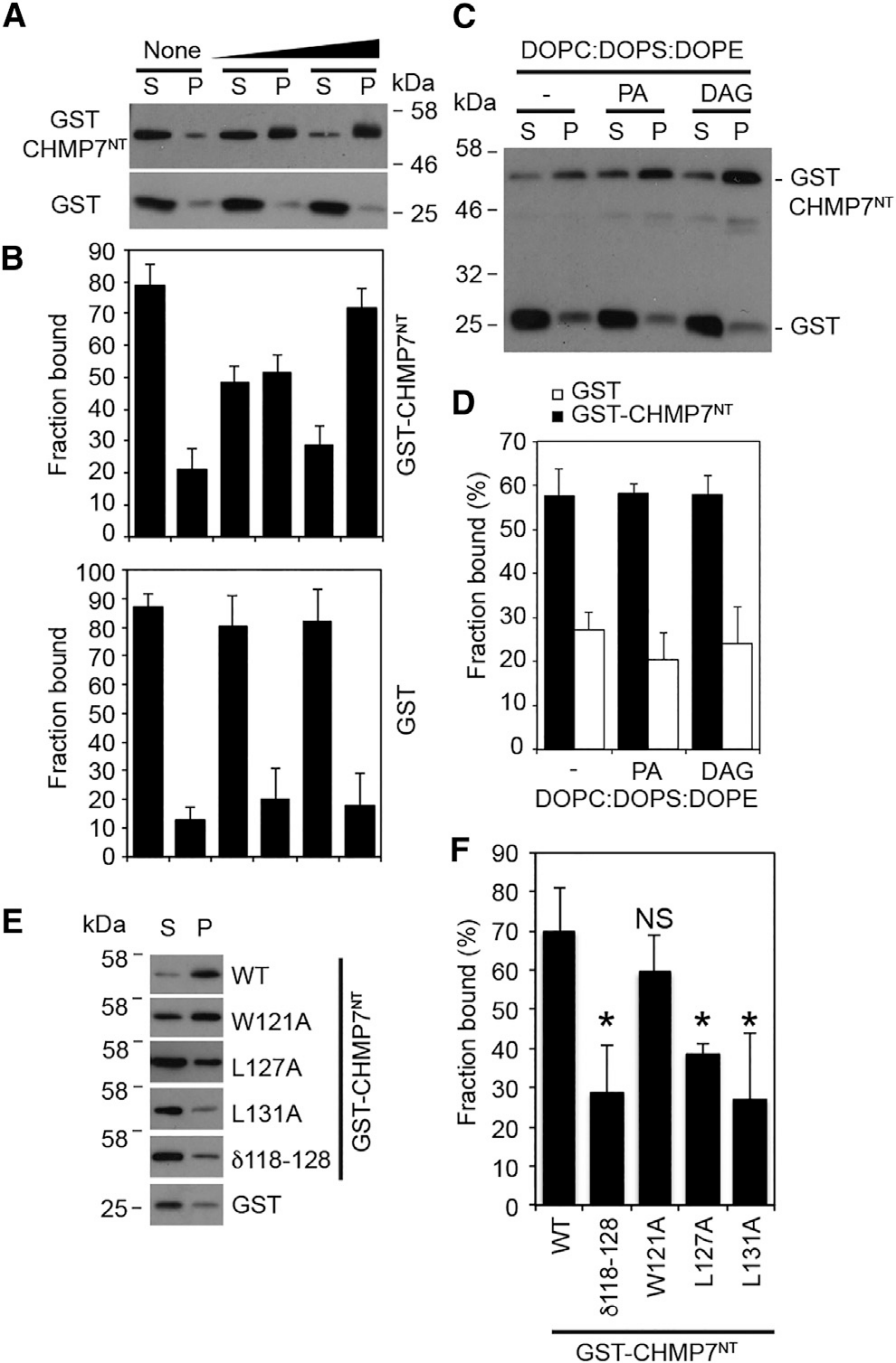
CHMP7^NT^ Binds Lipid Membranes GST or GST-CHMP7^NT^ was incubated for 5 min (A and B) or 15 min (C–F) with Folch (A, B, E, and F) or synthetic (C and D; 60% 1,2-dioleoyl-sn-glycero-3-phosphocholine [DOPC], 20% 1,2-dioleoyl-sn-glycero-3-phosphoserine [DOPS], and 20% 1,2-dioleoyl-sn-glycero-3-phosphoethanolamine [DOPE]) liposomes. In (A), increasing amounts (0, 10, or 50 μg) of liposomes were added. In (D), 2.5% 1-2-dioleoyl-*sn*-glycerol (DAG) or 1,2-dioleoyl-*sn*-glycero-3-phosphate (PA) was added, as indicated. Liposomes were collected by ultracentrifugation. Pelleted (P) and soluble (S) fractions were resolved and analyzed by western blotting with anti-GST antisera. Western blots from (A), (C), and (E) were quantified by densitometry (mean ± SD) (B and D, N = 3; F, WT, N = 7; δ118–128, N = 7; W121A, N = 7, NS; L127A, N = 4; L131A, N = 4). Statistical significance was calculated using one-way ANOVA with Dunnett’s multiple comparison test (^∗^p < 0.0001). See also Figure S4.

We wondered whether the hydrophobic residues necessary for ER localization acted as a membrane-binding region to anchor this protein in the ER, and we found that HIS-CHMP7^NT^ and GST-CHMP7^NT^, but not GST, could be captured upon liposomes (Figures 3A–3D and S4A). The fusogenic lipid diacylglycerol has been implicated in NE reformation [15], however, membrane interaction of CHMP7^NT^ was insensitive to the presence of diacylglycerol (Figures 3C and 3D). We also found membrane interaction to be insensitive to the degree of membrane curvature (Figures S4B and S4C). Mutation of residues that disrupted ER localization prevented membrane binding, with deletion of the hydrophobic cluster (δ118–128) or mutation of L127A or L131A having the strongest effect (Figures 3E and 3F). Importantly, these mutations did not destabilize GST-CHMP7^NT^ (Figure S4D).

**Figure 4:**
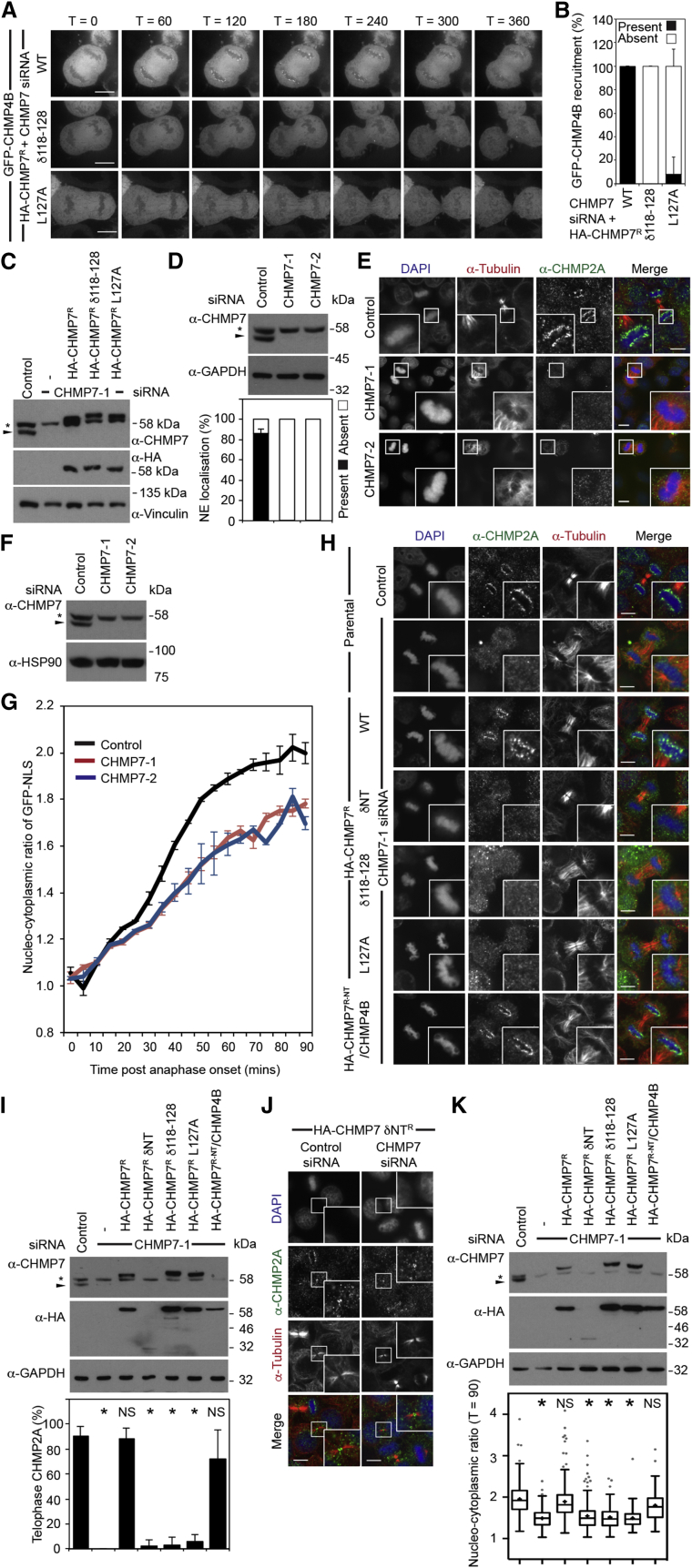
Membrane Binding by CHMP7 Is Essential for ESCRT-III-Dependent NE Reformation during Mitotic Exit (A) siRNA-transfected HeLa cells stably expressing both GFP-CHMP4B and the indicated HA-CHMP7^R^ proteins were imaged live (time interval in seconds). (B) Quantification of NE enrichment of GFP-CHMP4B from (A) (mean ± SD; CHMP7 siRNA + HA-CHMP7^R^, 23/23, N = 4; CHMP7 siRNA and HA-CHMP7^R^ δ118–128, 0/15, N = 3; CHMP7 siRNA and HA-CHMP7^R^ L127A, 1/16, N = 3). (C) Resolved lysates of cells from (A) were examined by western blotting with anti-CHMP7, anti-HA, or anti-Vinculin. (D and E) siRNA-transfected HeLa cells were fixed; stained with anti-tubulin, anti-CHMP2A, and DAPI; and examined by immunofluorescence (E) or resolved and examined by western blotting with anti-CHMP7 or anti-GAPDH (D). Assembly of CHMP2A at the telophase NE was quantified (D, mean ± SD; n = 40, N = 2; p = 0.0008, calculated by two-tailed Student’s t test). (F and G) siRNA-transfected HeLa cells stably expressing GFP-NLS and H2B-mCh were analyzed by western blotting with anti-CHMP7 and anti-HSP90 (F) or were imaged live and the degree of nucleocytoplasmic compartmentalization was calculated at the indicated time points (G, Mean ± SEM; control, N = 4, n = 40; CHMP7 siRNA-1, N = 3, p = 0.010, n = 28; CHMP7 siRNA-2, N = 3, n = 28, p = 0.003). Significance was calculated after 90 min using one-way ANOVA with Dunnett’s multiple comparison test. (H and I) HeLa cells stably expressing the indicated HA-tagged, siRNA-resistant CHMP7 proteins were transfected with control or CHMP7-targeting siRNA and fixed; stained with anti-tubulin, anti-CHMP2A, and DAPI; and examined by immunofluorescence (H) or resolved and analyzed by western blotting with anti-CHMP7, anti-HA, and anti-GAPDH (I). Cells from (H) were quantified (I, mean ± SD; control, N = 5, n = 80; CHMP7 siRNA, N = 4, n = 34, p < 0.001; CHMP7 siRNA and HA-CHMP7^R^, N = 4, n = 52, not significant [NS, p = 0.995]; CHMP7 siRNA and HA-CHMP7^R^ δNT, N = 4, n = 40, p < 0.001; CHMP7 siRNA and HA-CHMP7^R^ δ118–128, N = 3, n = 30, p < 0.001; CHMP7 siRNA and HA-CHMP7^R^ L127A, N = 3, n = 32, p < 0.001; CHMP7 siRNA and HA-CHMP7^R-NT^/CHMP4B, N = 3, n = 31, NS [p = 0.055]). Significance was calculated using one-way ANOVA with Dunnett’s multiple comparison test (^∗^, significant). (J) HeLa cells stably expressing HA-CHMP7^R^ δNT were transfected with control or CHMP7-targeting siRNA; fixed; stained with anti-tubulin, anti-CHMP2A, and DAPI; and examined by immunofluorescence. Midbody localization of CHMP2A was observed in 30/30 cases (Ctrl) and 29/30 cases (CHMP7 siRNA) (N = 3). (K) siRNA-transfected HeLa cells stably expressing GFP-NLS, H2B-mCh, and the indicated HA-tagged siRNA-resistant CHMP7 proteins were examined by western blotting with anti-CHMP7, anti-HA, or anti-GAPDH or were imaged live and the degree of nucleocytoplasmic compartmentalization was calculated 90 min post-anaphase onset (mean ± SEM; control, 1.93 ± 0.04, N = 9, n = 236; CHMP7 siRNA, 1.51 ± 0.03, N = 9, n = 252, p < 0.0001; CHMP7 siRNA and HA-CHMP7^R^, 1.88 ± 0.05, N = 8, n = 257, NS [p = 0.8909]; CHMP7 siRNA and HA-CHMP7^R^ δNT, 1.54 ± 0.08, N = 4, n = 207, p = < 0.0001; CHMP7 siRNA and HA-CHMP7^R^ δ118–128, 1.53 ± 0.1, N = 3, n = 101, p = 0.0003; CHMP7 siRNA and HA-CHMP7^R^ L127A, 1.50 ± 0.08, N = 3, n = 101, p = 0.0001; CHMP7 siRNA and HA-CHMP7^R-NT^/CHMP4B, 1.73 ± 0.1, N = 3, n = 76, NS [p = 0.1556]). Statistical significance was calculated using one-way ANOVA with Dunnett’s multiple comparison test from experimental means (N); ^∗^, significant. Tukey whiskers and mean (+) are displayed. In all micrographs, the scale bar represents 10 μm.

Consistent with a role for CHMP7 in recruiting CHMP4 proteins to the reforming NE (Figures S2F and S2G) [3], we found that stable expression of siRNA-resistant HA-CHMP7 (HA-CHMP7^R^), but neither HA-CHMP7^R^ δ118–128 nor HA-CHMP7^R^ L127A, could support enrichment of GFP-CHMP4B at the reforming NE in CHMP7-depleted cells (Figures 4A–4C; Movie S4). Further, we found that CHMP7 depletion prevented enrichment of endogenous CHMP2A at the reforming NE (Figures 4D and 4E). Failure to recruit CHMP2A to this organelle leaves holes in the reforming NE [2], and, consistent with this (and [5]), we found that CHMP7 depletion led to a poorly sealed post-mitotic NE (Figures 4F and 4G). Assembly of CHMP2A at the reforming NE in CHMP7-depleted cells could be rescued by stable expression of HA-CHMP7^R^ or HA-CHMP7^NT-R^/CHMP4B, but not by HA-CHMP7^R^ δNT, HA-CHMP7^R^ δ118–128, or HA-CHMP7^R^ L127A (Figures 4H and 4I). Midbody accumulation of endogenous CHMP2A was unaffected in CHMP7-depleted cells expressing HA-CHMP7^R^ δNT (Figure 4J), indicating that the membrane-binding ability of CHMP7 is required for NE-specific ESCRT-III function. While the nucleo-cytoplasmic compartmentalization defect induced by CHMP7 depletion could be rescued by stable expression of HA-CHMP7^R^, stable expression of HA-CHMP7^R^ δNT, HA-CHMP7^R^ δ118–128, or HA-CHMP7^R^ L127A failed to rescue this compartmentalization defect (Figure 4K). Just as the chimeric CHMP7^NT-R^/CHMP4B could support ESCRT-III assembly at the reforming NE, it could rescue the nucleo-cytoplasmic compartmentalization defect elicited by CHMP7 depletion (Figure 4K).

We describe a membrane-binding domain that localizes CHMP7 to the ER and, given the continuity of the ER with the NE [16], its subsequent function in regenerating a sealed NE during mitotic exit. Consistent with a role for ESCRT-II in recruiting ESCRT-III to cellular membranes [17, 18, 19], the ESCRT-II-like N terminus of CHMP7 directs ESCRT-III assembly at the NE. In *C. elegans*, ESCRT-II has been reported to localize to the sarcoplasmic reticulum, suggesting that the tandem WH fold may play a broader role in ER targeting [20]. We identify specific residues in the first WH domain of CHMP7^NT^ domain that are necessary for membrane binding, ER localization, subsequent enrichment of CHMP7 at the reforming NE, and, given CHMP7’s ability to bind CHMP4 proteins [6, 11], that are essential for the assembly of downstream ESCRT-III components and for ESCRT-III-dependent NE regeneration. In the absence of membrane-bound CHMP7, ESCRT-III cannot assemble at the NE. CHMP7^NT^ was not stabilized at the reforming NE, suggesting that subsequent engagement of ESCRT-III [11] (and ESCRT-III-binding partners such as Spastin [3] or UFD1L [2]) by the C terminus of CHMP7 provides a stabilizing cue.

Recent reports describing the association of CHMP7’s C terminus with LEM family proteins [21] and that LEMD2 depletion impairs ESCRT-III assembly at this organelle [22] indicate that these also may be candidates that regulate the enrichment of CHMP7 at sites of annular fusion. While CHMP7’s membrane binding was curvature insensitive, a geometric constraint of the narrow-radius hole that is to be closed also may restrict subsequent ESCRT-III assembly to these sites. This geometric constraint may allow for a critical concentration of ESCRT-III components to be reached for productive filament assembly at the NE rather than the ER. CHMP7 is thus an ER-specific membrane adaptor for ESCRT-III that provides an activity essential for post-mitotic organelle biogenesis, and it may be necessary for repair of the NE under physiological and pathological conditions, such as in cancer or during migration-induced rupture [4, 5, 23].

## Author Contributions

J.G.C. conceived and designed the study. J.G.C., A.P.-R., and Y.O. performed data acquisition, analysis, and interpretation. J.G.C. and Y.O. drafted and revised the manuscript.

## Supplemental Information

Document S1. Supplemental Experimental Procedures and Figures S1–S4 Document S1. Supplemental Experimental Procedures and Figures S1–S4

Movie S1. CHMP7 Localizes to the ER and Is Enriched at the Reforming NE Concatenated time-lapse movies of: mNG-CHMP7 edited CAL-51 cells (clone 4A4 and clone 10A10) imaged live through the anaphase-telophase transition (representative of 17/17 acquired movies from all clones analysed); HeLa cells stably expressing GFPCHMP7 and imaged live through the anaphase-telophase transition (representative of 21/21 acquired movies); Cos7 cells stably expressing GFP-CHMP7 and imaged live through the anaphase-telophase transition (representative of 5/5 acquired movies); Cos7 cells stably expressing GFP-CHMP7 and imaged live during interphase (representative of 72/72 captured live cells); HeLa cells stably expressing GFP-CHMP7 were transfected with a plasmid encoding BIP-mCh-KDEL and imaged live through the anaphase-telophase transition (representative of 3/3 acquired movies). In all cases, frames were acquired every 30 seconds and displayed at 10 frames per second. Movie S1. CHMP7 Localizes to the ER and Is Enriched at the Reforming NE

Movie S2. CHMP7’s NT Determines ER Localization Concatenated time-lapse movies of: HeLa cells stably expressing GFP-CHMP7^NT^ and imaged live through the anaphase-telophase transition (representative of 22/22 acquired movies); HeLa cells stably expressing GFP-CHMP7^NT^, transfected with a plasmid encoding BIP-mCh-KDEL and imaged live through the anaphase-telophase transition (representative of 3/3 acquired movies); HeLa cells stably expressing GFP-CHMP7 δNT and imaged live through the anaphase-telophase transition (movie representative of 5/5 acquired movies). In all cases, frames were acquired every 30 seconds and displayed at 10 frames per second. Movie S2. CHMP7’s NT Determines ER Localization

Movie S3. A Hydrophobic Cluster in WH1 of CHMP7’s NT Determines ER Localization Concatenated time-lapse movies of: HeLa cells expressing the indicated GFP-CHMP7 proteins (GFP-CHMP7 δ118-128, GFP-CHMP7 W118A, GFP-CHMP7 W121A, GFP-CHMP7 F126A, GFP-CHMP7 L127A, GFP-CHMP7 L131A) and imaged live through the anaphase-telophase transition. In all cases, movie representative of 3/3 acquired movies. Frames were acquired every 30 seconds and displayed at 10 frames per second. Movie S3. A Hydrophobic Cluster in WH1 of CHMP7’s NT Determines ER Localization

Movie S4. Membrane Binding by CHMP7’s NT Is Essential for the Assembly of Downstream ESCRT-III Components at the Reforming NE Concatenated time-lapse movies of: HeLa cells stably expressing GFP-CHMP4B or both GFP-CHMP4B and the indicated HACHMP7^R^ proteins and imaged live through the anaphase-telophase transition. Cells were treated with Control siRNA or CHMP7-targeting siRNA as indicated. NE enrichment of GFP-CHMP4B was supported in 23/23 imaged cells (HA-CHMP7^R^, N = 4), 0/15 cells (HA-CHMP7^R^ δ118-128, N = 3), or 1/16 cells (HA-CHMP7^R^ L127A, N = 3). Frames were acquired every 30 seconds and displayed at 10 frames per second. Movie S4. Membrane Binding by CHMP7’s NT Is Essential for the Assembly of Downstream ESCRT-III Components at the Reforming NE

Document S2. Article plus Supplemental Information Document S2. Article plus Supplemental Information
