# Supplementary figures and images for: Membrane Binding by CHMP7 Coordinates ESCRT-III-Dependent Nuclear Envelope Reformation
Source: Curr Biol. 2016 Oct 10;26(19):2635–41. doi: 10.1016/j.cub.2016.07.039 (PMC5069351; doi:10.1016/j.cub.2016.07.039)

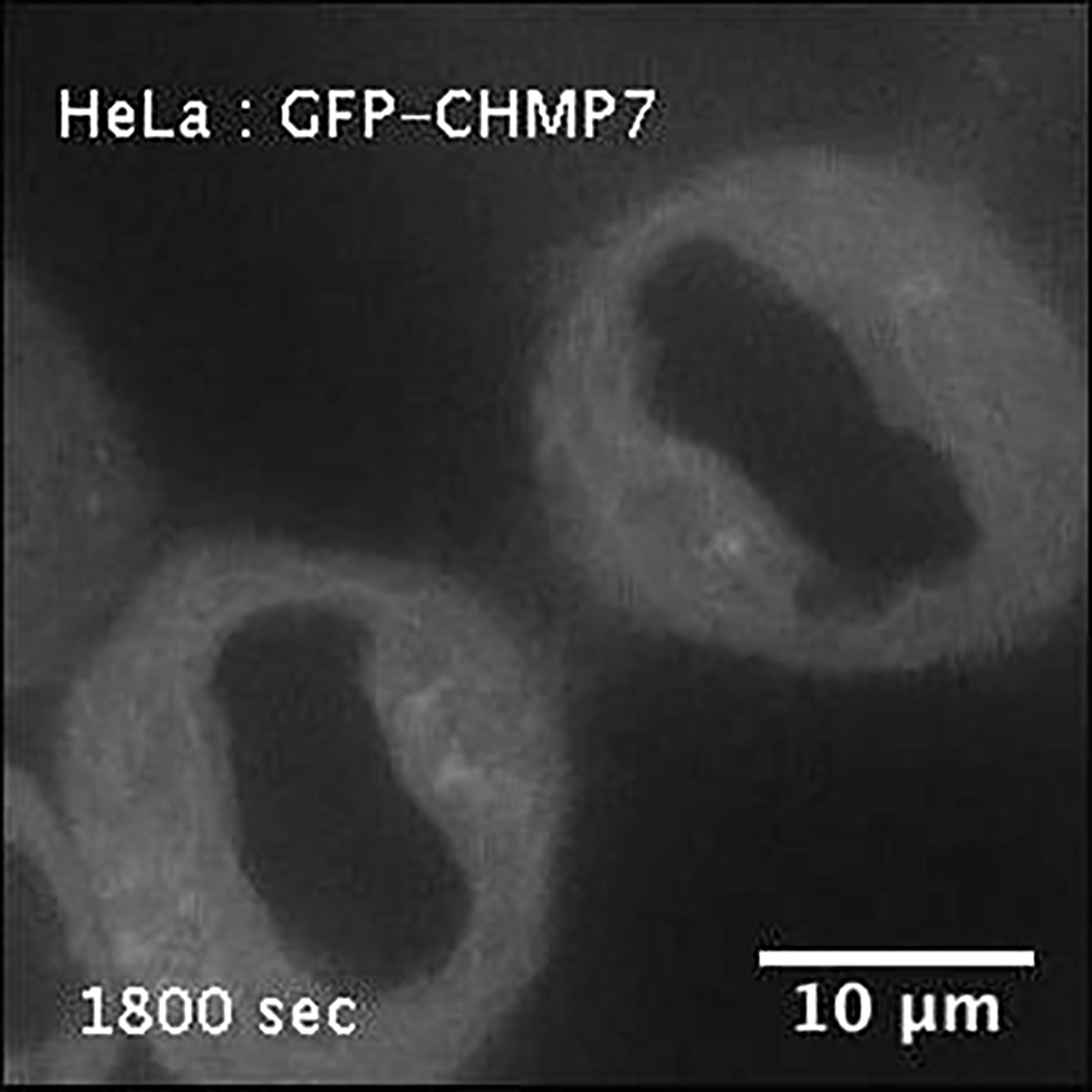

Supplement: Movie S1. CHMP7 Localizes to the ER and Is Enriched at the Reforming NE — Concatenated time-lapse movies of: mNG-CHMP7 edited CAL-51 cells (clone 4A4 and clone 10A10) imaged live through the anaphase-telophase transition (representative of 17/17 acquired movies from all clones analysed); HeLa cells stably expressing GFPCHMP7 and imaged live through the anaphase-telophase transition (representative of 21/21 acquired movies); Cos7 cells stably expressing GFP-CHMP7 and imaged live through the anaphase-telophase transition (representative of 5/5 acquired movies); Cos7 cells stably expressing GFP-CHMP7 and imaged live during interphase (representative of 72/72 captured live cells); HeLa cells stably expressing GFP-CHMP7 were transfected with a plasmid encoding BIP-mCh-KDEL and imaged live through the anaphase-telophase transition (representative of 3/3 acquired movies). In all cases, frames were acquired every 30 seconds and displayed at 10 frames per second. [file mmc2.jpg]

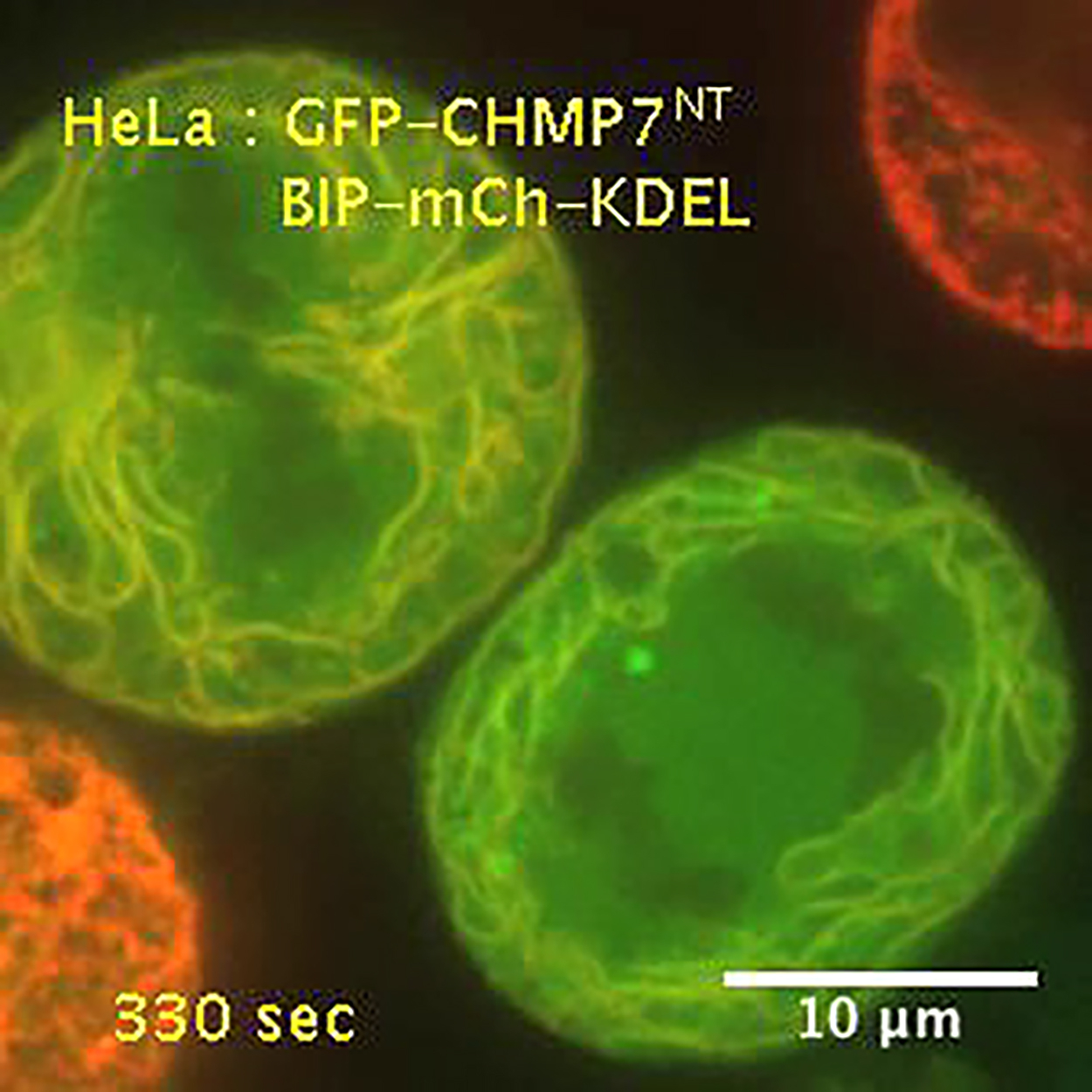

Supplement: Movie S2. CHMP7’s NT Determines ER Localization — Concatenated time-lapse movies of: HeLa cells stably expressing GFP-CHMP7NT and imaged live through the anaphase-telophase transition (representative of 22/22 acquired movies); HeLa cells stably expressing GFP-CHMP7NT, transfected with a plasmid encoding BIP-mCh-KDEL and imaged live through the anaphase-telophase transition (representative of 3/3 acquired movies); HeLa cells stably expressing GFP-CHMP7 δNT and imaged live through the anaphase-telophase transition (movie representative of 5/5 acquired movies). In all cases, frames were acquired every 30 seconds and displayed at 10 frames per second. [file mmc3.jpg]

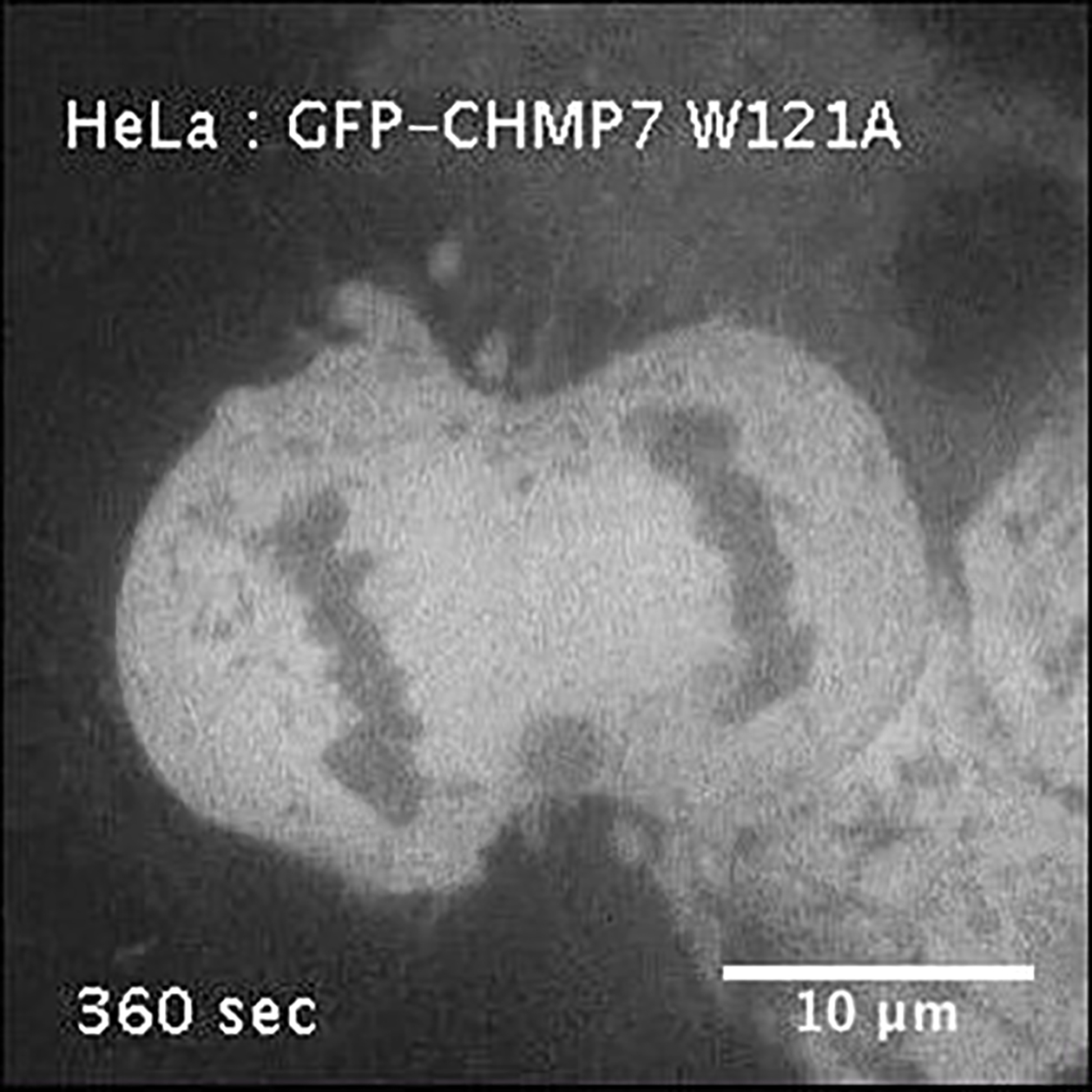

Supplement: Movie S3. A Hydrophobic Cluster in WH1 of CHMP7’s NT Determines ER Localization — Concatenated time-lapse movies of: HeLa cells expressing the indicated GFP-CHMP7 proteins (GFP-CHMP7 δ118-128, GFP-CHMP7 W118A, GFP-CHMP7 W121A, GFP-CHMP7 F126A, GFP-CHMP7 L127A, GFP-CHMP7 L131A) and imaged live through the anaphase-telophase transition. In all cases, movie representative of 3/3 acquired movies. Frames were acquired every 30 seconds and displayed at 10 frames per second. [file mmc4.jpg]

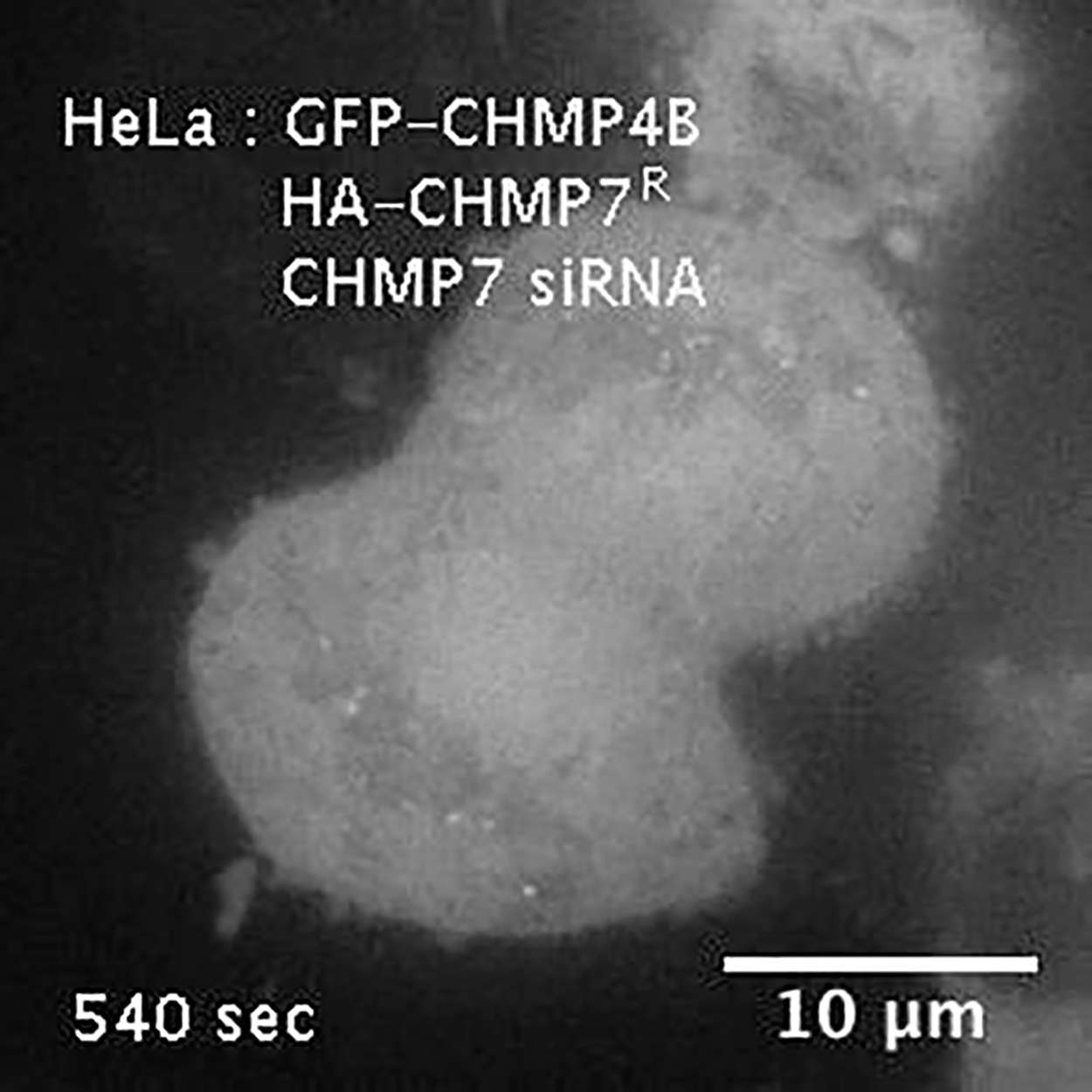

Supplement: Movie S4. Membrane Binding by CHMP7’s NT Is Essential for the Assembly of Downstream ESCRT-III Components at the Reforming NE — Concatenated time-lapse movies of: HeLa cells stably expressing GFP-CHMP4B or both GFP-CHMP4B and the indicated HACHMP7R proteins and imaged live through the anaphase-telophase transition. Cells were treated with Control siRNA or CHMP7-targeting siRNA as indicated. NE enrichment of GFP-CHMP4B was supported in 23/23 imaged cells (HA-CHMP7R, N = 4), 0/15 cells (HA-CHMP7R δ118-128, N = 3), or 1/16 cells (HA-CHMP7R L127A, N = 3). Frames were acquired every 30 seconds and displayed at 10 frames per second. [file mmc5.jpg]
