# Supplementary material for: Membrane Binding by CHMP7 Coordinates ESCRT-III-Dependent Nuclear Envelope Reformation
Source: Curr Biol. 2016 Oct 10;26(19):2635–41. doi: 10.1016/j.cub.2016.07.039 (PMC5069351; doi:10.1016/j.cub.2016.07.039)
Supplement: Document S2. Article plus Supplemental Information [file mmc6.pdf]

Current Biology

Report

Membrane Binding by CHMP7 Coordinates ESCRT-III-Dependent Nuclear Envelope Reformation

Graphical Abstract

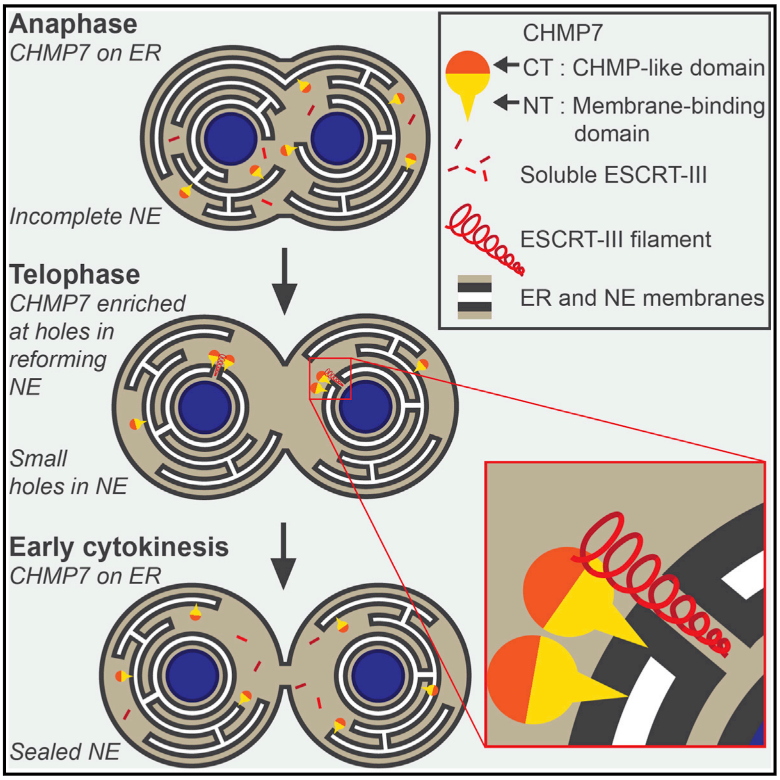

Authors

Yolanda Olmos, Anna Perdrix-Rosell, Jeremy G. Carlton

Correspondence

jeremy.carlton@kcl.ac.uk

In Brief

ESCRT-III is necessary for closing small holes in the reforming nuclear envelope during mitotic exit. Olmos et al. show that CHMP7, a key component of this machinery, contains a novel membrane-binding domain that is necessary for the assembly of ESCRT-III at this organelle and for the regeneration of a sealed nuclear envelope during mitotic exit.

Highlights

- CHMP7's N terminus is a membrane-binding domain
- Membrane binding allows CHMP7 to localize to the ER
- ER binding is necessary for CHMP7 localization to the reforming NE
- CHMP7's membrane binding is necessary for regeneration of the post-mitotic NE

CrossMark

Olmos et al., 2016, Current Biology *26*, 2635–2641
October 10, 2016 © 2016 The Author(s). Published by Elsevier Ltd.
http://dx.doi.org/10.1016/j.cub.2016.07.039

CellPress

Current Biology
Report

Membrane Binding by CHMP7 Coordinates ESCRT-III-Dependent Nuclear Envelope Reformation

Yolanda Olmos,[1] Anna Perdrix-Rosell,[1,2] and Jeremy G. Carlton[1,3,*]
[1]Division of Cancer Studies, King's College London, London SE1 1UL, UK
[2]Present address: Tumour-Host Interaction Laboratory, The Francis Crick Institute, Lincoln's Inn Fields Laboratory, London WC2A 3LY, UK
[3]Lead Contact
*Correspondence: jeremy.carlton@kcl.ac.uk
http://dx.doi.org/10.1016/j.cub.2016.07.039

SUMMARY

In addition to its role in membrane abscission during cytokinesis, viral budding, endosomal sorting, and plasma membrane repair [1], the endosomal sorting complex required for transport-III (ESCRT-III) machinery has recently been shown to seal holes in the reforming nuclear envelope (NE) during mitotic exit [2, 3]. ESCRT-III also acts during interphase to repair the NE upon migration-induced rupture [4, 5], highlighting its key role as an orchestrator of membrane integrity at this organelle. While NE localization of ESCRT-III is dependent upon the ESCRT-III component CHMP7 [3], it is unclear how this complex is able to engage nuclear membranes. Here we show that the N terminus of CHMP7 acts as a novel membrane-binding module. This membrane-binding ability allows CHMP7 to bind to the ER, an organelle continuous with the NE, and it provides a platform to direct NE recruitment of ESCRT-III during mitotic exit. CHMP7's N terminus comprises tandem Winged-Helix domains [6], and, by using homology modeling and structure-function analysis, we identify point mutations that disrupt membrane binding and prevent both ER localization of CHMP7 and its subsequent enrichment at the reforming NE. These mutations also prevent assembly of downstream ESCRT-III components at the reforming NE and proper establishment of post-mitotic nucleocytoplasmic compartmentalization. These data identify a novel membrane-binding activity within an ESCRT-III subunit that is essential for post-mitotic nuclear regeneration.

RESULTS AND DISCUSSION

CHMP7 is unique among endosomal sorting complex required for transport-III (ESCRT-III) subunits in that it contains an extended N terminus (NT) (Figures 1A and S1A) that we hypothesized may be important during its role in nuclear envelope (NE) regeneration. Using a dual-nickase CRISPR/Cas9 approach [7], we edited the CHMP7 locus in CAL-51 cells to produce a homozygous N-terminal fusion of monomeric-NeonGreen (mNG) [8] to CHMP7 under the control of its endogenous promoter (Figures 1B and S1B–S1I). These cells grew normally, suggesting that N-terminal tagging of CHMP7 is benign. We imaged living mNG-CHMP7 cells and found that, while CHMP7 was recruited to the NE during mitotic exit, in addition to a cytoplasmic pool, it decorated ER membranes in interphase and mitotic cells (Figures 1C and S1J; Movie S1). Stably expressed GFP-CHMP7 localized similarly (Figures 1D–1F and S1K–S1M). We saw no localization of GFP-CHMP7 to the midbody (Figure S1N). CHMP7-antisera failed to detect small interfering RNA (siRNA)-sensitive immunofluorescence signal; however, we could detect endogenous CHMP7 in ER fractions from homogenized cells (Figures 1G and S1O).

S. cerevisiae Chm7 was recently shown to localize to the ER [6], suggesting that this localization is evolutionarily conserved. During NE reformation, all other ESCRT-III subunits are recruited from the cytoplasm [2, 3]; given that the NE is formed from the ER [9, 10], a pre-existing ER localization for CHMP7 suggested a platform from which this recruitment could occur. Analysis of HeLa cells stably expressing GFP-CHMP7^{NT} or mCh-CHMP7^{NT} revealed that CHMP7's N terminus directed localization to the ER, but this truncated protein exhibited little stabilization at the reforming NE (Figures 1D and S2A–S2D; Movie S2). In contrast, the C terminus of CHMP7 (GFP-CHMP7 δNT) was cytosolic and displayed neither ER localization nor stabilization at the reforming NE (Figure 1H; Movie S2), despite containing the CHMP4B/ESCRT-III interaction domain [11]. CHMP7 is responsible for recruiting downstream ESCRT-III components to the reforming NE through CHMP4B. Fusion of siRNA-resistant CHMP7^{NT} to CHMP4B directed cytoplasmic CHMP4B to the mitotic ER and restored its enrichment at sites of annular fusion at the forming NE, in the absence of endogenous CHMP7 (Figures S2E–S2G).

Analysis of the secondary structure of CHMP7^{NT} has revealed the presence of tandem winged helix (WH) domains [6, 12], resembling those found in ESCRT-II subunits (Figure S3A). During endosomal sorting, membrane-anchored ESCRT-II serves to recruit ESCRT-III to endosomes through interaction of the second WH domain of VPS25 with the ESCRT-III component VPS20/CHMP6 [13, 14]. As CHMP7 initiates ESCRT-III assembly at the NE, we wondered whether its N terminus acted as a membrane adaptor at this organelle. HHpred (https://toolkit.tuebingen.mpg.de/hhpred) alignments of CHMP7 matched its NT to VPS25 [6], and, by aligning predicted secondary structural elements in CHMP7 to those present in the crystal structure of VPS25, we noted an evolutionarily conserved extension of the

This is an open access article under the CC BY license (http://creativecommons.org/licenses/by/4.0/).

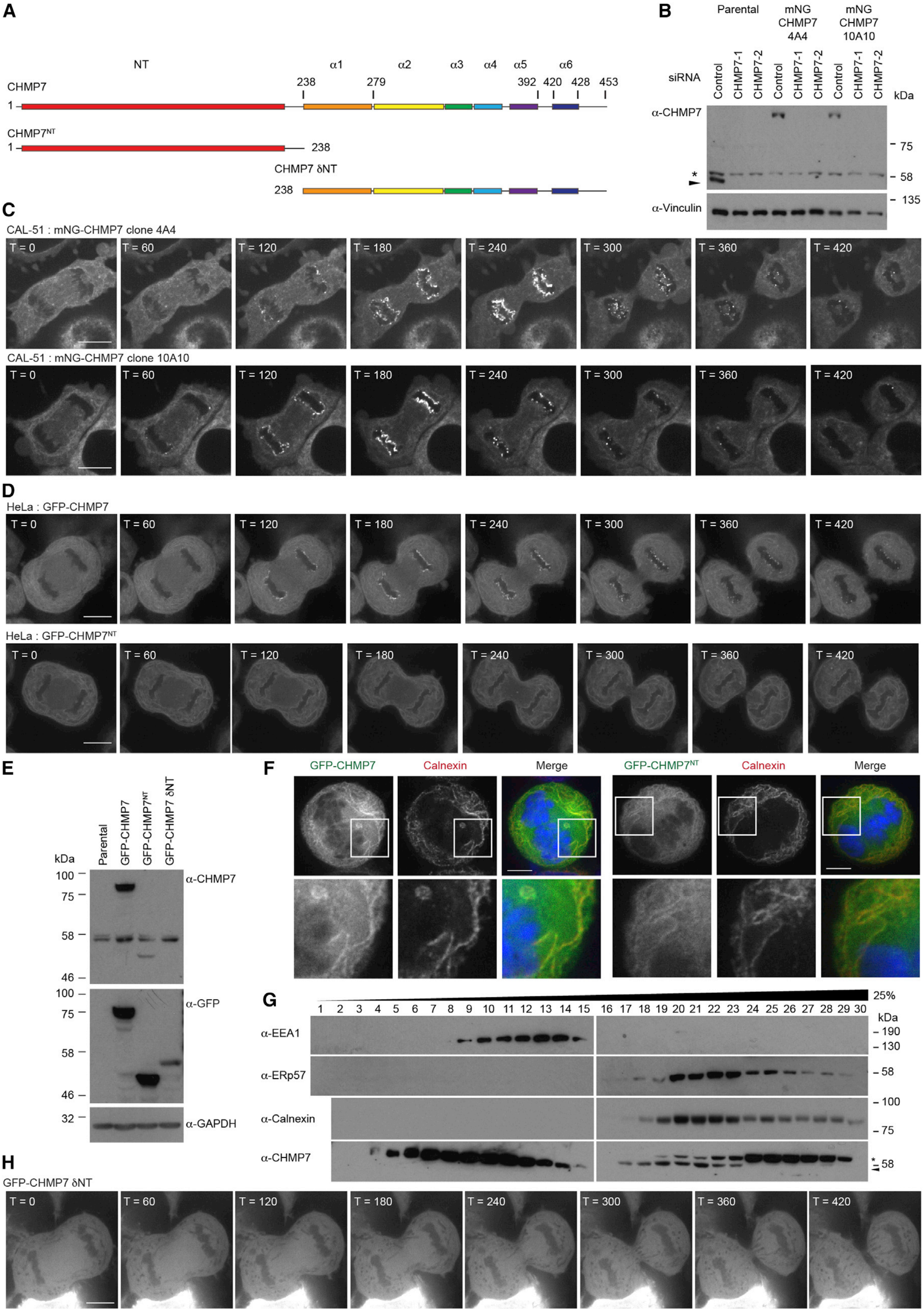

(legend on next page)

loop between the β2-β3 hairpin in the first WH domain of CHMP7^{NT} (Figure S3A).

Deletions through CHMP7^{NT} were poorly expressed (Figures S2C and S2D), so we performed scanning mutagenesis through CHMP7^{NT} to identify ER localization determinants (Figures S3B and S3C). We discovered 12 mutagenic tetrads that prevented ER localization, five of which were found on the extended loop in CHMP7^{NT}-WH1. We created a homology model of CHMP7^{NT} (lacking the extended loop) and mapped the remaining mutations to regions that were either in or engaged with residues on the WH1 β2-β3 hairpin (Figure S3D). Deletion of this loop (CHMP7 δ107–148) prevented ER localization (Figure 2A). In case alanine changes in blocks of four prevented proper folding, we mutated individual residues within this loop to fine-map determinants of ER localization. Mutation of six evolutionarily conserved hydrophobic residues (W118, W121, F126, L127, L128, and L131) or deletion of this hydrophobic stretch (δ118–128) prevented ER localization of CHMP7^{NT} (Figures 2A and 2B). These mutations prevented full-length CHMP7 from localizing to the ER and becoming enriched at the reforming NE (Figure 2C; Movie S3).

We wondered whether the hydrophobic residues necessary for ER localization acted as a membrane-binding region to anchor this protein in the ER, and we found that HIS-CHMP7^{NT} and GST-CHMP7^{NT}, but not GST, could be captured upon liposomes (Figures 3A–3D and S4A). The fusogenic lipid diacylglycerol has been implicated in NE reformation [15], however, membrane interaction of CHMP7^{NT} was insensitive to the presence of diacylglycerol (Figures 3C and 3D). We also found membrane interaction to be insensitive to the degree of membrane curvature (Figures S4B and S4C). Mutation of residues that disrupted ER localization prevented membrane binding, with deletion of the hydrophobic cluster (δ118–128) or mutation of L127A or L131A having the strongest effect (Figures 3E and 3F). Importantly, these mutations did not destabilize GST-CHMP7^{NT} (Figure S4D).

Consistent with a role for CHMP7 in recruiting CHMP4 proteins to the reforming NE (Figures S2F and S2G) [3], we found that stable expression of siRNA-resistant HA-CHMP7 (HA-CHMP7^{R}), but neither HA-CHMP7^{R} δ118–128 nor HA-CHMP7^{R} L127A, could support enrichment of GFP-CHMP4B at the reforming NE in CHMP7-depleted cells (Figures 4A–4C; Movie S4). Further, we found that CHMP7 depletion prevented enrichment of endogenous CHMP2A at the reforming NE (Figures 4D and 4E). Failure to recruit CHMP2A to this organelle leaves holes in the reforming NE [2], and, consistent with this (and [5]), we found that CHMP7 depletion led to a poorly sealed post-mitotic NE (Figures 4F and 4G). Assembly of CHMP2A at the reforming NE in CHMP7-depleted cells could be rescued by stable expression of HA-CHMP7^{R} or HA-CHMP7$^{NT\text{-}R}$/CHMP4B, but not by HA-CHMP7^{R} δNT, HA-CHMP7^{R} δ118–128, or HA-CHMP7^{R} L127A (Figures 4H and 4I). Midbody accumulation of endogenous CHMP2A was unaffected in CHMP7-depleted cells expressing HA-CHMP7^{R} δNT (Figure 4J), indicating that the membrane-binding ability of CHMP7 is required for NE-specific ESCRT-III function. While the nucleo-cytoplasmic compartmentalization defect induced by CHMP7 depletion could be rescued by stable expression of HA-CHMP7^{R}, stable expression of HA-CHMP7^{R} δNT, HA-CHMP7^{R} δ118–128, or HA-CHMP7^{R} L127A failed to rescue this compartmentalization defect (Figure 4K). Just as the chimeric CHMP7$^{NT\text{-}R}$/CHMP4B could support ESCRT-III assembly at the reforming NE, it could rescue the nucleo-cytoplasmic compartmentalization defect elicited by CHMP7 depletion (Figure 4K).

We describe a membrane-binding domain that localizes CHMP7 to the ER and, given the continuity of the ER with the NE [16], its subsequent function in regenerating a sealed NE during mitotic exit. Consistent with a role for ESCRT-II in recruiting ESCRT-III to cellular membranes [17–19], the ESCRT-II-like N terminus of CHMP7 directs ESCRT-III assembly at the NE. In *C. elegans*, ESCRT-II has been reported to localize to the sarcoplasmic reticulum, suggesting that the tandem WH fold may play a broader role in ER targeting [20]. We identify specific residues in the first WH domain of CHMP7^{NT} domain that are necessary for membrane binding, ER localization, subsequent enrichment of CHMP7 at the reforming NE, and, given CHMP7's ability to bind CHMP4 proteins [6, 11], that are essential for the assembly of downstream ESCRT-III components and for ESCRT-III-dependent NE regeneration. In the absence of membrane-bound CHMP7, ESCRT-III cannot assemble at the NE. CHMP7^{NT} was not stabilized at the reforming NE, suggesting that subsequent engagement of ESCRT-III [11] (and ESCRT-III-binding partners such as Spastin [3] or UFD1L [2]) by the C terminus of CHMP7 provides a stabilizing cue.

Recent reports describing the association of CHMP7's C terminus with LEM family proteins [21] and that LEMD2 depletion impairs ESCRT-III assembly at this organelle [22] indicate that these also may be candidates that regulate the enrichment of CHMP7 at sites of annular fusion. While CHMP7's membrane binding was curvature insensitive, a geometric constraint of the narrow-radius hole that is to be closed also may restrict

Figure 1. CHMP7 Is an ER-Localized Protein that Is Enriched at the NE during Mitotic Exit

(A) Schematic depicting constructs used in this study.

(B and C) CAL-51 cells edited to express mNG-CHMP7 were resolved and examined by western blotting with anti-CHMP7 and anti-Vinculin (B) or imaged live (C). In this and all other figures, endogenous CHMP7 is marked by an arrowhead (*, non-specific band).

(D–F) HeLa cells stably expressing GFP-CHMP7 and GFP-CHMP7^{NT} were imaged live (D), lysed, resolved, and examined by western blotting with anti-GFP, anti-CHMP7, or anti-GAPDH antisera (E) or fixed and stained with anti-Calnexin and DAPI (F). Images in (D) are representative of all cells imaged and 22/22 (GFP-CHMP7) and 21/21 (GFP-CHMP7^{NT}) captured movies. Co-localization of GFP-CHMP7 and GFP-CHMP7^{NT} with Calnexin was observed in 7/7 and 13/13 scored cells, respectively.

(G) Post-nuclear supernatants from Cos7 cells were fractionated through a continuous iodixanol gradient and analyzed by western blotting with the indicated antisera.

(H) HeLa cells stably expressing GFP-CHMP7 δNT were imaged live. Images are representative of all cells imaged and 5/5 captured movies. Time interval is presented in seconds post-cortical ingression.

In all micrographs, the scale bar represents 10 μm. See also Figure S1.

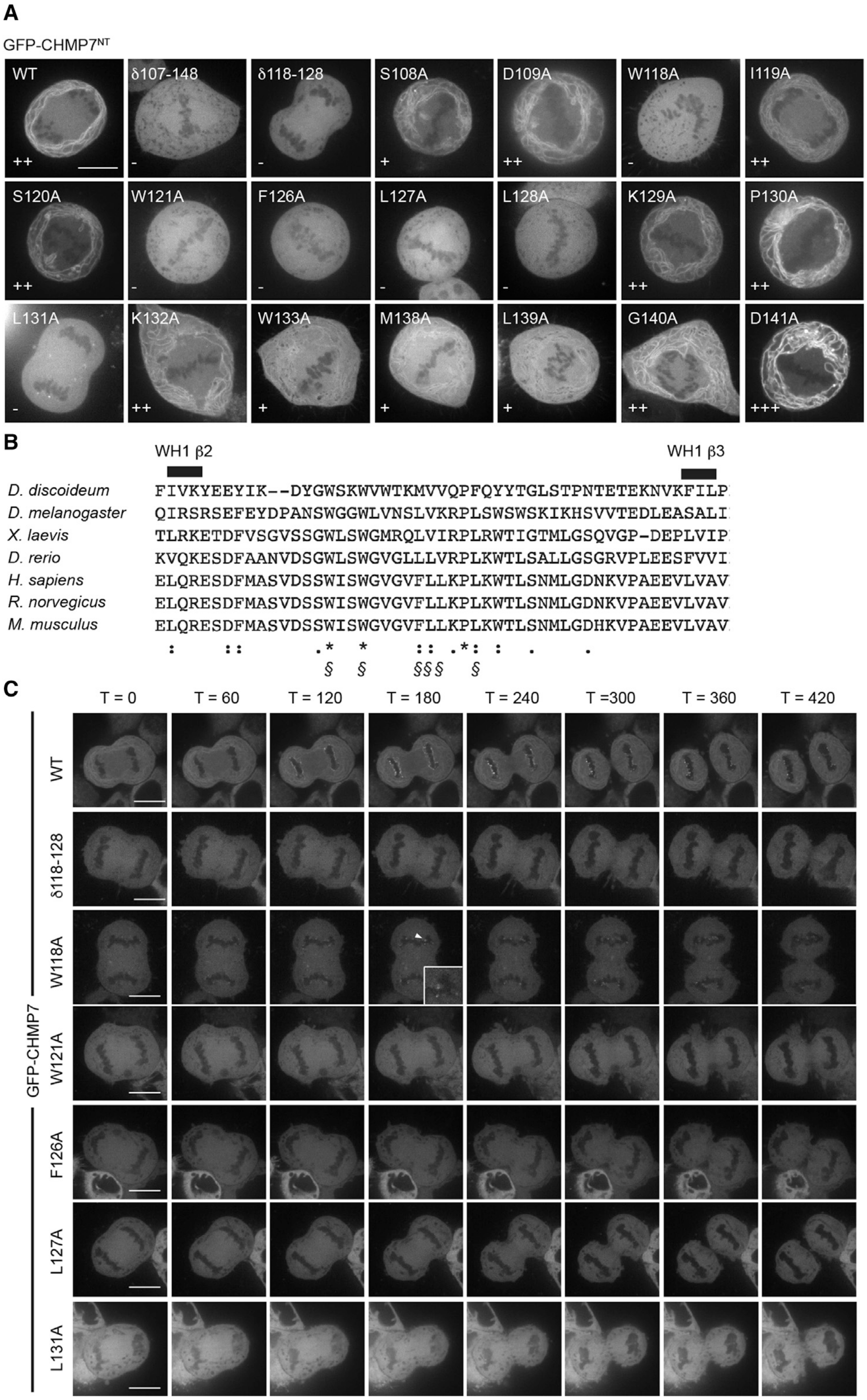

Figure 2. Mapping Activities in CHMP7^{NT} that Govern ER Localization

(A) HeLa cells were transfected with the indicated GFP-CHMP7^{NT} plasmids and imaged live; individual residues within the β2-β3 insertion that disrupted ER localization when previously mutated in blocks of four (Figures S3B–S3D) were mutated to alanine. Reticular localization is indicated as follows: –, none/cytoplasmic; +, reduced; ++, normal; and +++, enhanced). Reticular localization was observed in the indicated number of captured images from three independent experiments. NT, 11/11; δ107–148, 0/11; δ 118–128, 0/13; S108A, 12/12; D109A, 11/11; W118A, 0/13; I119A, 11/11; S120A, 11/11; W121A,

(legend continued on next page)

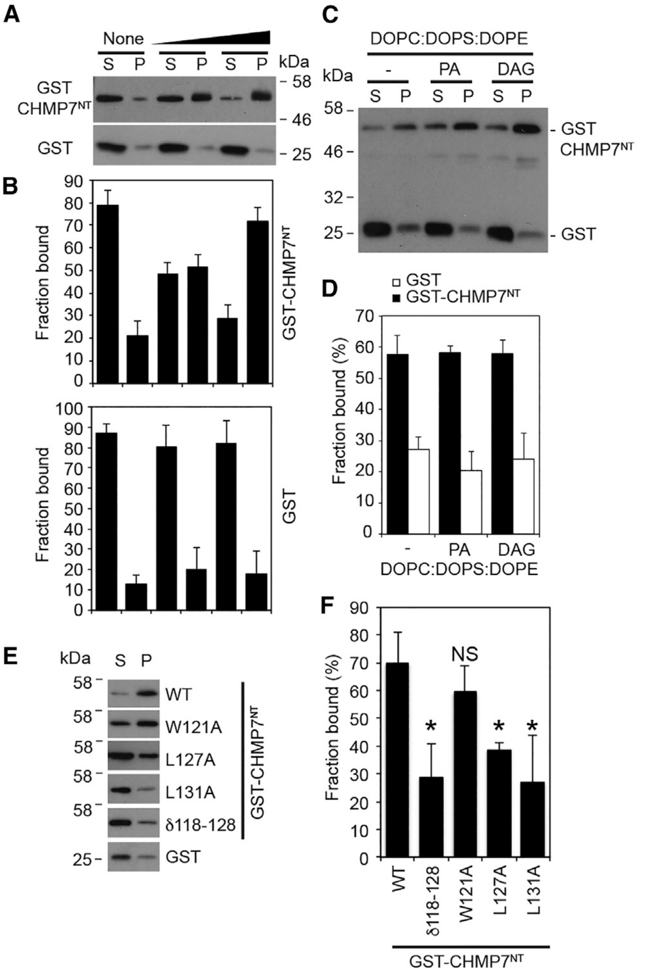

Figure 3. CHMP7^{NT} Binds Lipid Membranes

GST or GST-CHMP7^{NT} was incubated for 5 min (A and B) or 15 min (C–F) with Folch (A, B, E, and F) or synthetic (C and D; 60% 1,2-dioleoyl-sn-glycero-3-phosphocholine [DOPC], 20% 1,2-dioleoyl-sn-glycero-3-phosphoserine [DOPS], and 20% 1,2-dioleoyl-sn-glycero-3-phosphoethanolamine [DOPE]) liposomes. In (A), increasing amounts (0, 10, or 50 μg) of liposomes were added. In (D), 2.5% 1-2-dioleoyl-*sn*-glycerol (DAG) or 1,2-dioleoyl-*sn*-glycero-3-phosphate (PA) was added, as indicated. Liposomes were collected by ultracentrifugation. Pelleted (P) and soluble (S) fractions were resolved and analyzed by western blotting with anti-GST antisera. Western blots from (A), (C), and (E) were quantified by densitometry (mean ± SD) (B and D, N = 3; F, WT, N = 7; δ118–128, N = 7; W121A, N = 7, NS; L127A, N = 4; L131A, N = 4). Statistical significance was calculated using one-way ANOVA with Dunnett's multiple comparison test (*$p < 0.0001$). See also Figure S4.

subsequent ESCRT-III assembly to these sites. This geometric constraint may allow for a critical concentration of ESCRT-III components to be reached for productive filament assembly at the NE rather than the ER. CHMP7 is thus an ER-specific membrane adaptor for ESCRT-III that provides an activity essential for post-mitotic organelle biogenesis, and it may be necessary for repair of the NE under physiological and pathological conditions, such as in cancer or during migration-induced rupture [4, 5, 23].

SUPPLEMENTAL INFORMATION

Supplemental Information includes Supplemental Experimental Procedures, four figures, and four movies and can be found with this article online at http://dx.doi.org/10.1016/j.cub.2016.07.039.

AUTHOR CONTRIBUTIONS

J.G.C. conceived and designed the study. J.G.C., A.P.-R., and Y.O. performed data acquisition, analysis, and interpretation. J.G.C. and Y.O. drafted and revised the manuscript.

ACKNOWLEDGMENTS

J.G.C. is a Wellcome Trust Research Career Development Fellow (093603/Z/10/Z). We acknowledge the Nikon Imaging Centre at KCL and the Programme in Infection and Immunity Flow Cytometry Core at KCL for access to equipment. We thank Camille Wouters and Nisreen Chahid who assisted with cloning as part of high school Nuffield Research Placements. We thank Dr. Pierfrancesco Marra (KCL) for guidance on density-gradient centrifugation.

Received: April 29, 2016
Revised: June 25, 2016
Accepted: July 15, 2016
Published: September 8, 2016

REFERENCES

1. Hurley, J.H. (2015). ESCRTs are everywhere. EMBO J. *34*, 2398–2407.
2. Olmos, Y., Hodgson, L., Mantell, J., Verkade, P., and Carlton, J.G. (2015). ESCRT-III controls nuclear envelope reformation. Nature *522*, 236–239.
3. Vietri, M., Schink, K.O., Campsteijn, C., Wegner, C.S., Schultz, S.W., Christ, L., Thoresen, S.B., Brech, A., Raiborg, C., and Stenmark, H. (2015). Spastin and ESCRT-III coordinate mitotic spindle disassembly and nuclear envelope sealing. Nature *522*, 231–235.
4. Raab, M., Gentili, M., de Belly, H., Thiam, H.R., Vargas, P., Jimenez, A.J., Lautenschlaeger, F., Voituriez, R., Lennon-Duménil, A.M., Manel, N., and Piel, M. (2016). ESCRT III repairs nuclear envelope ruptures during cell migration to limit DNA damage and cell death. Science *352*, 359–362.
5. Denais, C.M., Gilbert, R.M., Isermann, P., McGregor, A.L., te Lindert, M., Weigelin, B., Davidson, P.M., Friedl, P., Wolf, K., and Lammerding, J. (2016). Nuclear envelope rupture and repair during cancer cell migration. Science *352*, 353–358.
6. Bauer, I., Brune, T., Preiss, R., and Kölling, R. (2015). Evidence for a non-endosomal function of the Saccharomyces cerevisiae ESCRT-III-like protein Chm7. Genetics *201*, 1439–1452.
7. Cong, L., Ran, F.A., Cox, D., Lin, S., Barretto, R., Habib, N., Hsu, P.D., Wu, X., Jiang, W., Marraffini, L.A., and Zhang, F. (2013). Multiplex genome engineering using CRISPR/Cas systems. Science *339*, 819–823.

0/12; F126A, 0/12; L127A, 0/14; L128A, 0/14; K129A, 13/13; P130A, 13/13; L131A, 0/12; K132A, 12/13; W133A, 13/14; M138A, 15/15; L139A, 11/12; G140A, 16/16; D141A, 13/13.

(B) Sequence alignment of the insertion between β2 and β3 in the CHMP7^{NT} WH1 domain from the indicated organisms. Conservation extent is as follows: *, complete; :, strongly similar; •, weakly similar; §, residues necessary for ER localization.

(C) HeLa cells expressing the indicated GFP-CHMP7 constructs were imaged live (time in seconds post-cortical ingression). Images are representative of 3/3 acquired movies and 50/50 scored cells per mutation. Limited enrichment on the telophase NE (boxed) was observed for GFP-CHMP7 W118A, suggesting that some degree of ER localization persists in this case.

In all micrographs, the scale bar represents 10 μm. See also Figures S2 and S3.

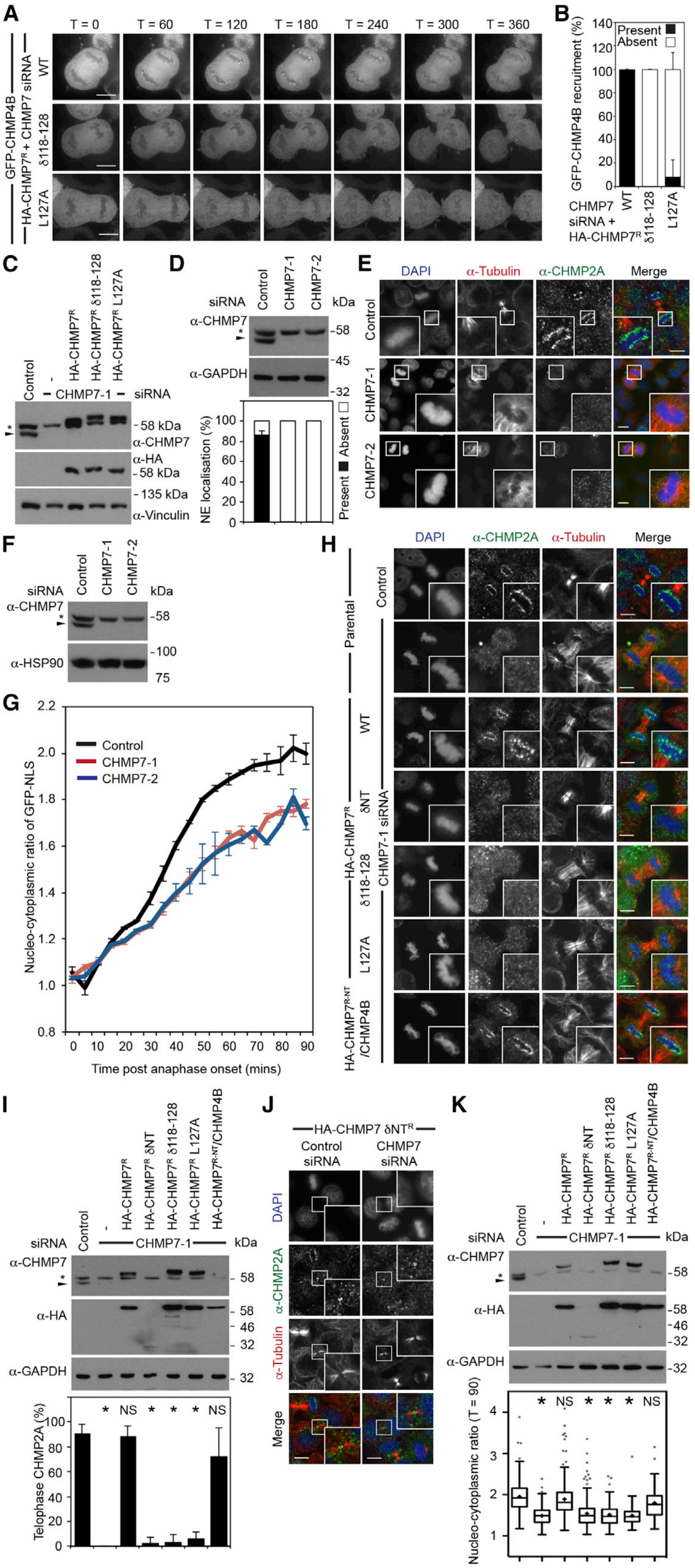

Figure 4. Membrane Binding by CHMP7 Is Essential for ESCRT-III-Dependent NE Reformation during Mitotic Exit

(A) siRNA-transfected HeLa cells stably expressing both GFP-CHMP4B and the indicated HA-CHMP7^R proteins were imaged live (time interval in seconds).

(B) Quantification of NE enrichment of GFP-CHMP4B from (A) (mean ± SD; CHMP7 siRNA + HA-CHMP7^R, 23/23, N = 4; CHMP7 siRNA and HA-CHMP7^R δ118–128, 0/15, N = 3; CHMP7 siRNA and HA-CHMP7^R L127A, 1/16, N = 3).

(C) Resolved lysates of cells from (A) were examined by western blotting with anti-CHMP7, anti-HA, or anti-Vinculin.

(D and E) siRNA-transfected HeLa cells were fixed; stained with anti-tubulin, anti-CHMP2A, and DAPI; and examined by immunofluorescence (E) or resolved and examined by western blotting with anti-CHMP7 or anti-GAPDH (D). Assembly of CHMP2A at the telophase NE was quantified (D, mean ± SD; n = 40, N = 2; p = 0.0008, calculated by two-tailed Student's t test).

(F and G) siRNA-transfected HeLa cells stably expressing GFP-NLS and H2B-mCh were analyzed by western blotting with anti-CHMP7 and anti-HSP90 (F) or were imaged live and the degree of nucleocytoplasmic compartmentalization was calculated at the indicated time points (G, Mean ± SEM; control, N = 4, n = 40; CHMP7 siRNA-1, N = 3, p = 0.010, n = 28; CHMP7 siRNA-2, N = 3, n = 28, p = 0.003). Significance was calculated after 90 min using one-way ANOVA with Dunnett's multiple comparison test.

(H and I) HeLa cells stably expressing the indicated HA-tagged, siRNA-resistant CHMP7 proteins were transfected with control or CHMP7-targeting siRNA and fixed; stained with anti-tubulin, anti-CHMP2A, and DAPI; and examined by immunofluorescence (H) or resolved and analyzed by western blotting with anti-CHMP7, anti-HA, and anti-GAPDH (I). Cells from (H) were quantified (I, mean ± SD; control, N = 5, n = 80; CHMP7 siRNA, N = 4, n = 34, p < 0.001; CHMP7 siRNA and HA-CHMP7^R, N = 4, n = 52, not significant [NS, p = 0.995]; CHMP7 siRNA and HA-CHMP7^R δNT, N = 4, n = 40, p < 0.001; CHMP7 siRNA and HA-CHMP7^R δ118–128, N = 3, n = 30, p < 0.001; CHMP7 siRNA and HA-CHMP7^R L127A, N = 3, n = 32, p < 0.001; CHMP7 siRNA and HA-CHMP7$^{R\text{-}NT}$/CHMP4B, N = 3, n = 31, NS [p = 0.055]). Significance was calculated using one-way ANOVA with Dunnett's multiple comparison test (*, significant).

(J) HeLa cells stably expressing HA-CHMP7^R δNT were transfected with control or CHMP7-targeting siRNA; fixed; stained with anti-tubulin, anti-CHMP2A, and DAPI; and examined by immunofluorescence. Midbody localization of CHMP2A was observed in 30/30 cases (Ctrl) and 29/30 cases (CHMP7 siRNA) (N = 3).

(K) siRNA-transfected HeLa cells stably expressing GFP-NLS, H2B-mCh, and the indicated HA-tagged siRNA-resistant CHMP7 proteins were examined by western blotting with anti-CHMP7, anti-HA, or anti-GAPDH or were imaged live and the degree of nucleocytoplasmic compartmentalization was calculated 90 min post-anaphase onset (mean ± SEM; control, 1.93 ± 0.04, N = 9, n = 236; CHMP7 siRNA, 1.51 ± 0.03, N = 9, n = 252, p < 0.0001; CHMP7 siRNA and HA-CHMP7^R, 1.88 ± 0.05, N = 8, n = 257, NS [p = 0.8909]; CHMP7 siRNA and HA-CHMP7^R δNT, 1.54 ± 0.08, N = 4, n = 207, p = < 0.0001; CHMP7 siRNA and HA-CHMP7^R δ118–128, 1.53 ± 0.1, N = 3, n = 101, p = 0.0003; CHMP7 siRNA and HA-CHMP7^R L127A, 1.50 ± 0.08, N = 3, n = 101, p = 0.0001; CHMP7 siRNA and HA-CHMP7$^{R\text{-}NT}$/CHMP4B, 1.73 ± 0.1, N = 3, n = 76, NS [p = 0.1556]). Statistical significance was calculated using one-way ANOVA with Dunnett's multiple comparison test from experimental means (N); *, significant. Tukey whiskers and mean (+) are displayed.

In all micrographs, the scale bar represents 10 μm.

8. Shaner, N.C., Lambert, G.G., Chammas, A., Ni, Y., Cranfill, P.J., Baird, M.A., Sell, B.R., Allen, J.R., Day, R.N., Israelsson, M., et al. (2013). A bright monomeric green fluorescent protein derived from Branchiostoma lanceolatum. Nat. Methods *10*, 407–409.

9. Lu, L., Ladinsky, M.S., and Kirchhausen, T. (2009). Cisternal organization of the endoplasmic reticulum during mitosis. Mol. Biol. Cell *20*, 3471–3480.

10. Anderson, D.J., and Hetzer, M.W. (2007). Nuclear envelope formation by chromatin-mediated reorganization of the endoplasmic reticulum. Nat. Cell Biol. *9*, 1160–1166.

11. Horii, M., Shibata, H., Kobayashi, R., Katoh, K., Yorikawa, C., Yasuda, J., and Maki, M. (2006). CHMP7, a novel ESCRT-III-related protein, associates with CHMP4b and functions in the endosomal sorting pathway. Biochem. J. *400*, 23–32.

12. Sundquist, W.I., and Ullman, K.S. (2015). CELL BIOLOGY. An ESCRT to seal the envelope. Science *348*, 1314–1315.

13. Teis, D., Saksena, S., Judson, B.L., and Emr, S.D. (2010). ESCRT-II coordinates the assembly of ESCRT-III filaments for cargo sorting and multivesicular body vesicle formation. EMBO J. *29*, 871–883.

14. Im, Y.J., Wollert, T., Boura, E., and Hurley, J.H. (2009). Structure and function of the ESCRT-II-III interface in multivesicular body biogenesis. Dev. Cell *17*, 234–243.

15. Domart, M.-C., Hobday, T.M.C., Peddie, C.J., Chung, G.H.C., Wang, A., Yeh, K., Jethwa, N., Zhang, Q., Wakelam, M.J.O., Woscholski, R., et al. (2012). Acute manipulation of diacylglycerol reveals roles in nuclear envelope assembly & endoplasmic reticulum morphology. PLoS ONE *7*, e51150.

16. Burke, B., and Ellenberg, J. (2002). Remodelling the walls of the nucleus. Nat. Rev. Mol. Cell Biol. *3*, 487–497.

17. Im, Y.J., and Hurley, J.H. (2008). Integrated structural model and membrane targeting mechanism of the human ESCRT-II complex. Dev. Cell *14*, 902–913.

18. Teo, H., Perisic, O., González, B., and Williams, R.L. (2004). ESCRT-II, an endosome-associated complex required for protein sorting: crystal structure and interactions with ESCRT-III and membranes. Dev. Cell *7*, 559–569.

19. Henne, W.M., Buchkovich, N.J., Zhao, Y., and Emr, S.D. (2012). The endosomal sorting complex ESCRT-II mediates the assembly and architecture of ESCRT-III helices. Cell *151*, 356–371.

20. Lefebvre, C., Largeau, C., Michelet, X., Fourrage, C., Maniere, X., Matic, I., Legouis, R., and Culetto, E. (2016). The ESCRT-II proteins are involved in shaping the sarcoplasmic reticulum in C. elegans. J. Cell Sci. *129*, 1490–1499.

21. Webster, B.M., Thaller, D.J., Jaeger, J., Ochmann, S.E., and Lusk, C.P. (2016). Chm7 and Heh1 form a nuclear envelope subdomain for nuclear pore complex quality control. bioRxiv. http://dx.doi.org/10.1101/049148.

22. Gu, M., Chen, O.S., Lajoie, D., Ladinsky, M.S., Redd, M.J., Nikolova, L., Bjorkman, P.J., Ullman, K.S., Sundquist, W.I., and Frost, A. (2016). LEM2 and CHMP7 function in ESCRT-dependent nuclear envelope closure in yeast and human cells. bioRxiv. http://dx.doi.org/10.1101/049312.

23. Vargas, J.D., Hatch, E.M., Anderson, D.J., and Hetzer, M.W. (2012). Transient nuclear envelope rupturing during interphase in human cancer cells. Nucleus *3*, 88–100.

Current Biology, Volume *26*

Supplemental Information

Membrane Binding by CHMP7 Coordinates ESCRT-III-Dependent Nuclear Envelope Reformation

Yolanda Olmos, Anna Perdrix-Rosell, and Jeremy G. Carlton

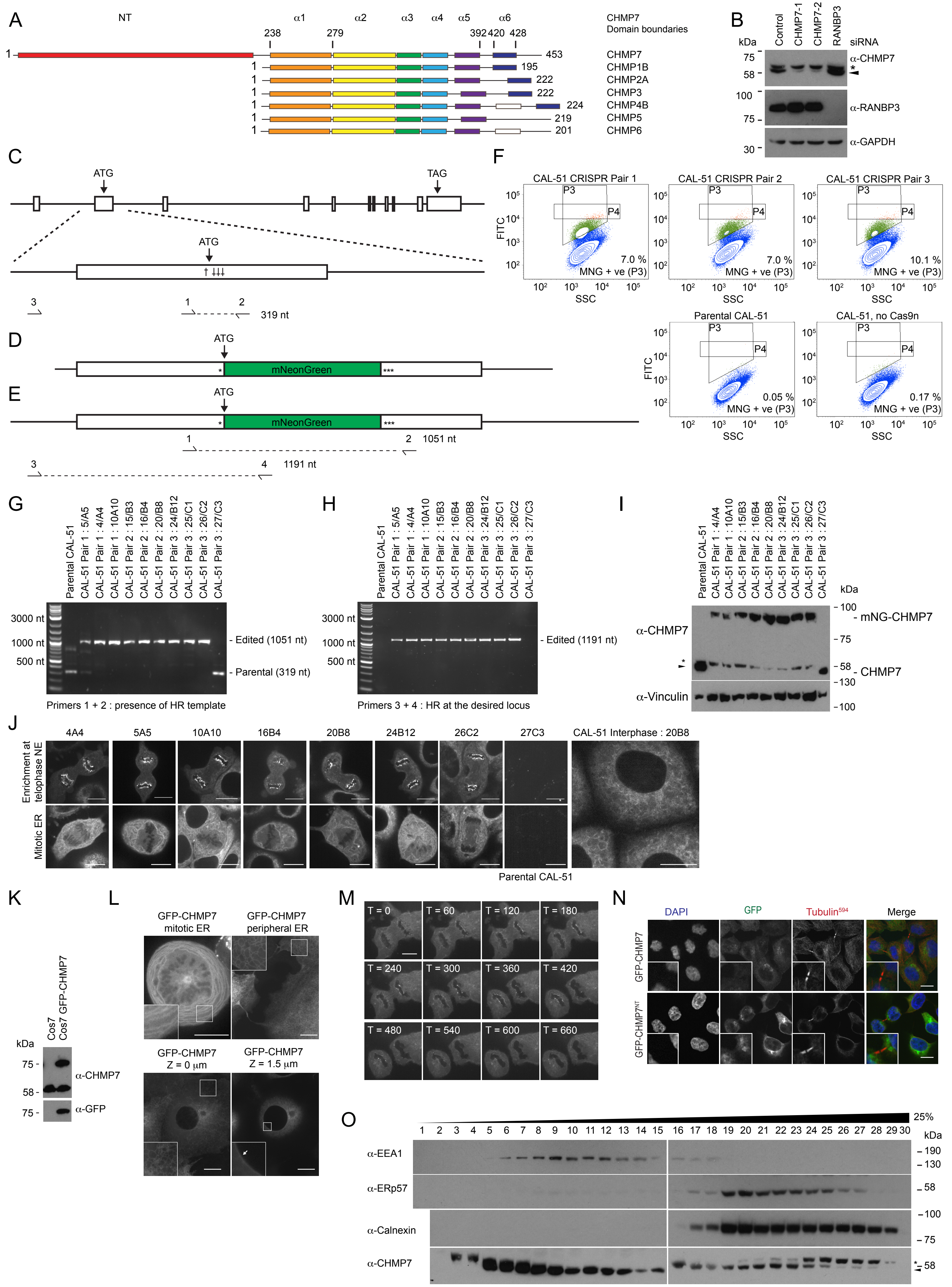
A
NT
α1
α2
α3
α4
α5
α6
CHMP7
Domain boundaries
238
279
392 420 428
453
195
222
222
224
219
201
CHMP7
CHMP1B
CHMP2A
CHMP3
CHMP4B
CHMP5
CHMP6
B
kDa
Control
CHMP7-1
CHMP7-2
RANBP3
siRNA
α-CHMP7
α-RANBP3
α-GAPDH
C
ATG
TAG
319 nt
D
mNeonGreen
E
1051 nt
1191 nt
F
CAL-51 CRISPR Pair 1
CAL-51 CRISPR Pair 2
CAL-51 CRISPR Pair 3
Parental CAL-51
CAL-51, no Cas9n
FITC
SSC
P3
P4
7.0 %
7.0 %
10.1 %
0.05 %
0.17 %
MNG + ve (P3)
G
H
I
Parental CAL-51
CAL-51 Pair 1 : 5/A5
CAL-51 Pair 1 : 4/A4
CAL-51 Pair 1 : 10A10
CAL-51 Pair 2 : 15/B3
CAL-51 Pair 2 : 16/B4
CAL-51 Pair 2 : 20/B8
CAL-51 Pair 3 : 24/B12
CAL-51 Pair 3 : 25/C1
CAL-51 Pair 3 : 26/C2
CAL-51 Pair 3 : 27/C3
3000 nt
1000 nt
500 nt
Edited (1051 nt)
Parental (319 nt)
Primers 1 + 2 : presence of HR template
Edited (1191 nt)
Primers 3 + 4 : HR at the desired locus
α-CHMP7
mNG-CHMP7
CHMP7
α-Vinculin
J
4A4
5A5
10A10
16B4
20B8
24B12
26C2
27C3
CAL-51 Interphase : 20B8
Enrichment at telophase NE
Mitotic ER
Parental CAL-51
K
Cos7
Cos7 GFP-CHMP7
α-CHMP7
α-GFP
L
GFP-CHMP7 mitotic ER
GFP-CHMP7 peripheral ER
GFP-CHMP7 Z = 0 μm
GFP-CHMP7 Z = 1.5 μm
M
T = 0
T = 60
T = 120
T = 180
T = 240
T = 300
T = 360
T = 420
T = 480
T = 540
T = 600
T = 660
N
DAPI
GFP
Tubulin594
Merge
GFP-CHMP7
GFP-CHMP7NT
O
25%
α-EEA1
α-ERp57
α-Calnexin
α-CHMP7

Figure S1, related to Figure 1 : CHMP7 localises to the ER

A. Domain analysis of CHMPs, diagram drawn from Bajorek et al., 2009 [S1]. B. To verify the siRNA-sensitive band detected by anti-CHMP7, resolved cell lysates from HeLa cells transfected with the indicated siRNAs were examined by western blotting with anti-CHMP7, anti-GAPDH or anti-RANBP3. In this, and all other figures, * is a non-specific band, endogenous CHMP7 indicated by arrowhead. C. Schematic showing the CHMP7 locus at 8p21.3, exons indicated by boxed regions and an enlargement of the exon containing CHMP7's start codon is provided. Within this exon, the position of selected guide RNAs indicated by dagger (forward) or inverted daggers (reverse). Primer pairs 1 and 2, designed to amplify a 319 nt region spanning the start codon indicated below. Primer 3 was designed to bind the 5' UTR. D. The HR template employed, * indicates mutations in residues comprising PAMs of the guide RNAs chosen. E. Predicted edited locus bearing HR-directed insertion of mNG-CHMP7 exon1, the new amplicon produced by primer pairs 1 and 2 (1051 nt) and a second amplicon created by the HR-mediated insertion that spans the repair template using primer pairs 3 and 4 (1191 nt). F. Flow-cytometric analysis of mNG fluorescence in CAL-51 cells 5 days after transfection. The percentage of mNG +ve cells given (P3). P4 represents the sort-gate for acquisition of single-cell clones. Cells were transfected with the HR-template and pairs of Cas9 D10A-linked guide RNAs, untransfected, or transfected with the HR template and no Cas9 D10A. G. PCR-based analysis of genomic DNA extracted from parental CAL-51 cells or edited clones to determine editing efficiency. 29/33 clones contained a homozygous insertion of the HR template, 1/33 clones contained a heterozygous insertion (5/A5), as judged by the presence of both parental and edited amplicons from the combination of primers 1 and 2. 3/33 clones contained no edit (e.g., 27/C3). Data for parental CAL-51 and 10 clones displayed. H. PCR-based analysis of genomic DNA extracted from clones to determine insertion in correct locus from PCR product spanning the insertion. In all edited cases from G, the expected amplicon was detected. I. Resolved lysates of selected clones of CAL-51 cells from S1H, edited to express mNG-CHMP7 were analysed by western blotting using anti-CHMP7 or anti-Vinculin. J. ER-localisation was observed in all interphase and mitotic cells in captured images from 27/29 individually imaged CAL-51 clones (451 scored interphase cells; 61 captured mitotic cells); clone 35C11 turned out to be a mixed population with un-edited cells and clone 21B9 died. Representative images of mitotic ER localisation and NE-enrichment given for the indicated clones presented in S1J, scale bar = 10 μm. Asynchronous fields of view given for parental and 27C3 (unedited) CAL-51 cells. K. Resolved cell lysates of Cos7 cells and Cos7 stably expressing GFP-CHMP7 at endogenous levels were analysed by western blotting with anti-CHMP7 and anti-GFP. L-M. Cos7 stably expressing GFP-CHMP7 were imaged live. Scale bar is 10 μm, images representative of all cells imaged and 72/72 captured interphase cells, 11/11 captured mitotic cells (L) and 5/5 captured movies of enrichment of GFP-CHMP7 at the reforming NE (M, time interval given in seconds). Note, GFP-CHMP7 was present on the interphase NE (arrows), but unlike during mitotic NE reformation, was not enriched upon this membrane compared to the ER (L). ER-localisation of GFP-CHMP7^{NT} was observed in all visualised and 19/19 captured interphase HeLa cells (data not shown). N. HeLa cells stably expressing GFP-CHMP7 or GFP-CHMP7^{NT} were fixed and stained with anti-tubulin and DAPI. Scale bar is 10 μm. O. Post-nuclear supernatants from HeLa cells were fractionated through a continuous iodixanol gradient and analysed by SDS-PAGE and western blotting with the indicated antisera.

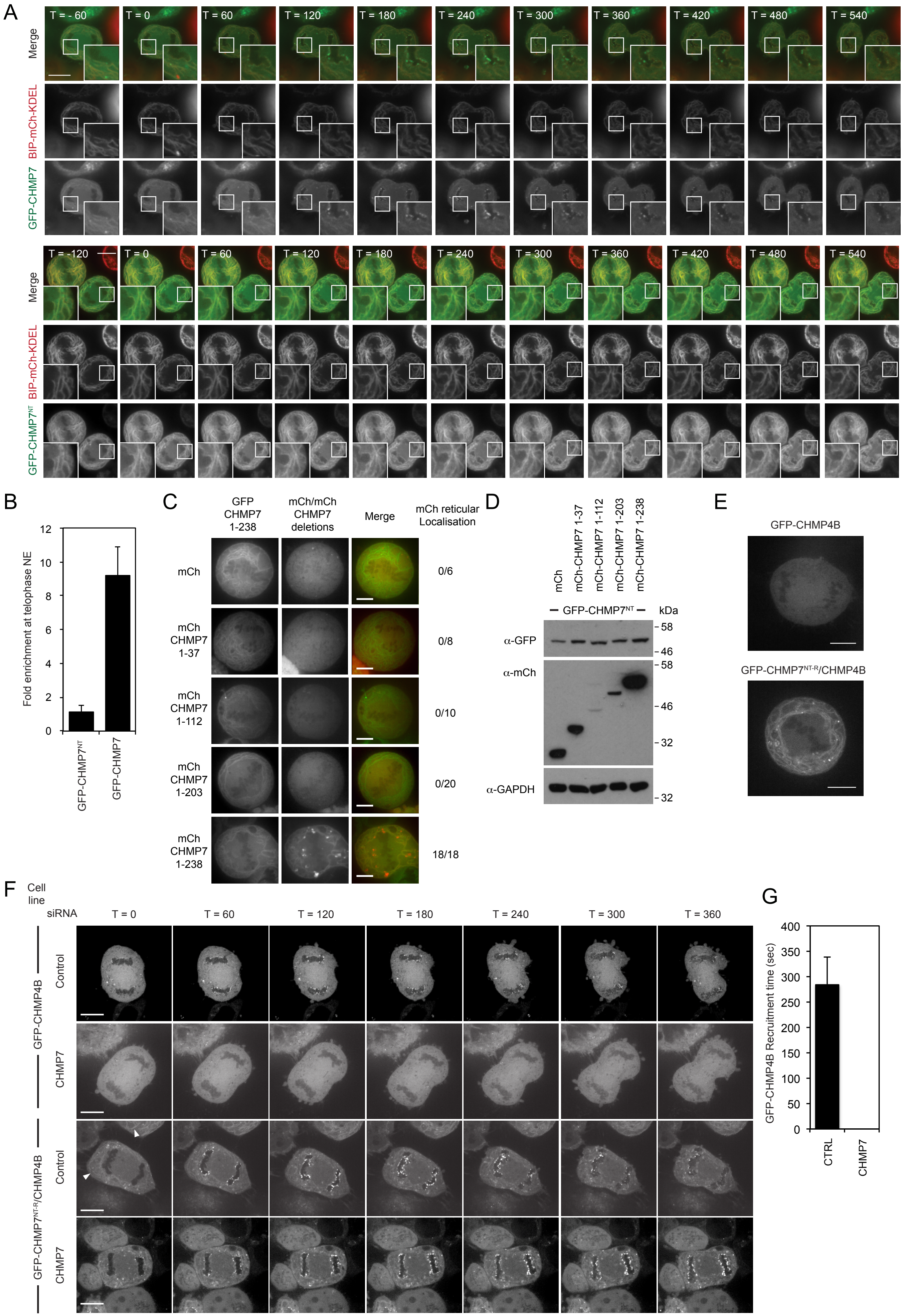
A
Merge
BIP-mCh-KDEL
GFP-CHMP7
T = - 60
T = 0
T = 60
T = 120
T = 180
T = 240
T = 300
T = 360
T = 420
T = 480
T = 540
T = -120
GFP-CHMP7NT
B
Fold enrichment at telophase NE
12
10
8
6
4
2
0
GFP-CHMP7NT
GFP-CHMP7
C
GFP CHMP7 1-238
mCh/mCh CHMP7 deletions
Merge
mCh reticular Localisation
mCh
0/6
mCh CHMP7 1-37
0/8
mCh CHMP7 1-112
0/10
mCh CHMP7 1-203
0/20
mCh CHMP7 1-238
18/18
D
mCh
mCh-CHMP7 1-37
mCh-CHMP7 1-112
mCh-CHMP7 1-203
mCh-CHMP7 1-238
GFP-CHMP7NT
kDa
α-GFP
α-mCh
α-GAPDH
58
46
58
46
32
32
E
GFP-CHMP4B
GFP-CHMP7NT-R/CHMP4B
F
Cell line
siRNA
T = 0
T = 60
T = 120
T = 180
T = 240
T = 300
T = 360
GFP-CHMP4B
Control
CHMP7
GFP-CHMP7NT-R/CHMP4B
Control
CHMP7
G
GFP-CHMP4B Recruitment time (sec)
400
350
300
250
200
150
100
50
0
CTRL
CHMP7

Figure S2, related to Figure 1 : CHMP7^{NT} directs ER localisation and is necessary for subsequent enrichment of CHMPs at the reforming NE.

A. HeLa cells stably expressing GFP-CHMP7 or GFP-CHMP7^{NT} were transfected with pLHCX BIP-mCh-KDEL and imaged live. Images representative of all cells imaged and 3/3 captured movies per condition. Scale bar is 10 µm, time in seconds post cortical ingression given. B. HeLa cells stably expressing GFP-CHMP7 or GFP-CHMP7^{NT} were imaged live and the degree of enrichment at the reforming NE was assessed by quantification of the maximal fluorescence intensity achieved at the NE compared to an adjacent region of ER (GFP-CHMP7, n = 10; GFP-CHMP7^{NT}, n = 4; mean ± S.D.) C. HeLa cells stably expressing both GFP-CHMP7^{NT} and the indicated mCh-CHMP7 deletions were imaged live and reticular localisation in mitotic cells was scored, scale bar is 10 µm. D. Resolved cell lysates from cells from C were examined by western blotting with anti-mCh, anti-GAPDH or anti-GFP. E. HeLa cells stably expressing GFP-CHMP4B or GFP-CHMP7^{NT}/CHMP4B were imaged live during mitosis. Images representative of all cells imaged and 5/5 captured cells per condition, scale bar is 10 µm. F. HeLa cells stably expressing GFP-CHMP4B or GFP-CHMP7^{NT-R}/CHMP4B were transfected with the indicated siRNA and imaged live. Images representative of 3/3 movies (GFP-CHMP4B, Control siRNA), 3/3 movies (GFP-CHMP4B, CHMP7 siRNA), 5/5 movies (GFP-CHMP7^{NT-R}/CHMP4B, Control siRNA), 6/6 movies (GFP-CHMP7^{NT-R}/CHMP4B , CHMP7 siRNA), arrowheads depict ER localisation, time given in seconds, , scale bar is 10 µm. G. Quantification of duration of GFP-CHMP4B localisation from movies in F (Duration presented in seconds ± S.D. Control, n = 6; CHMP7 siRNA n = 7).

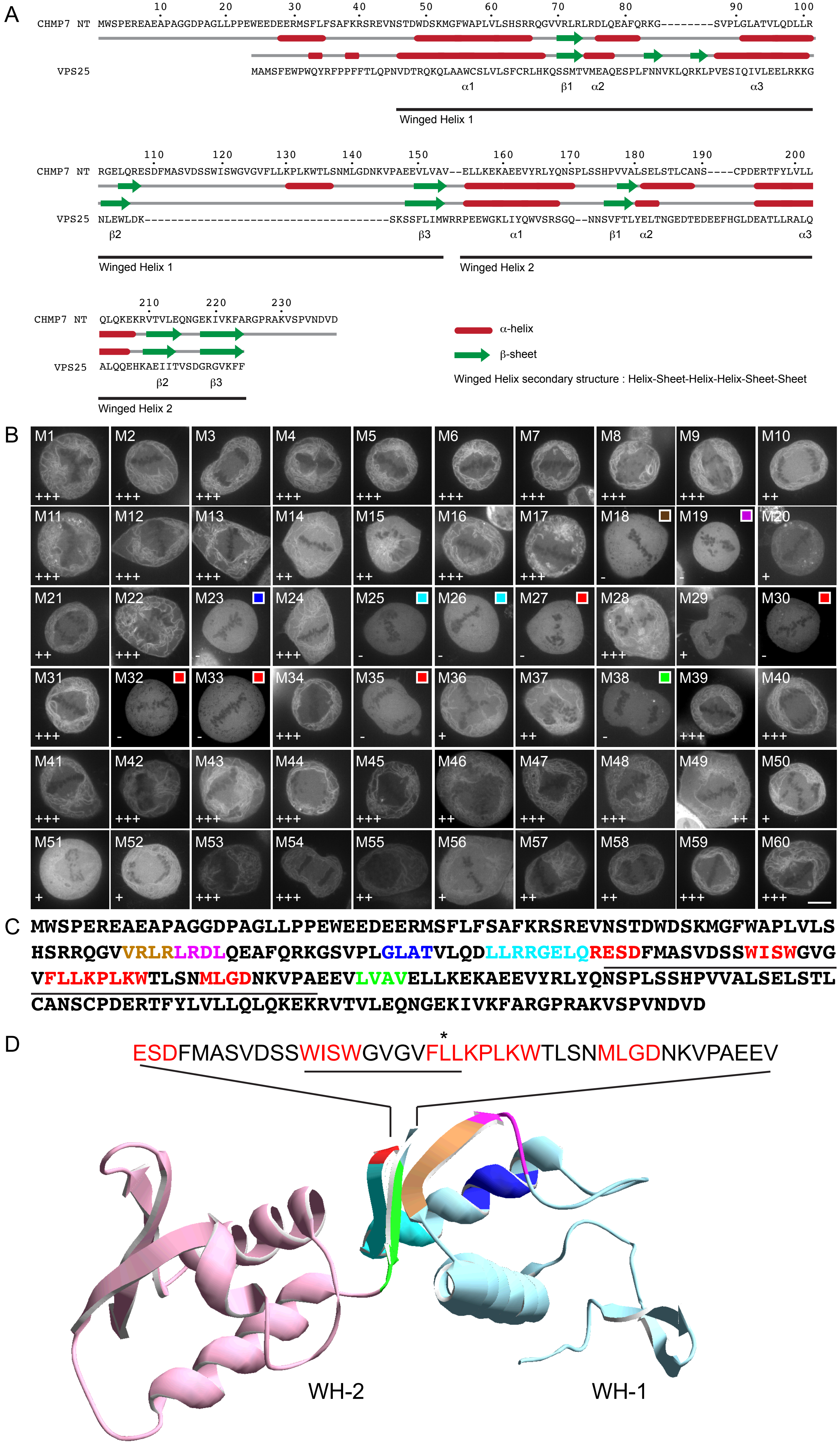
A
10 20 30 40 50 60 70 80 90 100
CHMP7 NT MWSPEREAEAPAGGDPAGLLPPEWEEDEERMSFLFSAFKRSREVNSTDWDSKMGFWAPLVLSHSRRQGVVRLRLRDLQEAFQRKG--------SVPLGLATVLQDLLR
VPS25 MAMSFEWPWQYRFPPFFTLQPNVDTRQKQLAAWCSLVLSFCRLHKQSSMTVMEAQESPLFNNVKLQRKLPVESIQIVLEELRKKG
α1 β1 α2 α3
Winged Helix 1
110 120 130 140 150 160 170 180 190 200
CHMP7 NT RGELQRESDFMASVDSSWISWGVGVFLLKPLKWTLSNMLGDNKVPAEEVLVAV--ELLKEKAEEVYRLYQNSPLSSHPVVALSELSTLCANS----CPDERTFYLVLL
VPS25 NLEWLDK-----------------------------------SKSSFLIMWRRPEEWGKLIYQWVSRSGQ--NNSVFTLYELTNGEDTEDEEFHGLDEATLLRALQ
β2 β3 α1 β1 α2 α3
Winged Helix 1
Winged Helix 2
210 220 230
CHMP7 NT QLQKEKRVTVLEQNGEKIVKFARGPRAKVSPVNDVD
VPS25 ALQQEHKAEIITVSDGRGVKFF
β2 β3
Winged Helix 2
α-helix
β-sheet
Winged Helix secondary structure : Helix-Sheet-Helix-Helix-Sheet-Sheet
B
M1 +++
M2 +++
M3 +++
M4 +++
M5 +++
M6 +++
M7 +++
M8 +++
M9 +++
M10 ++
M11 +++
M12 +++
M13 +++
M14 ++
M15 ++
M16 +++
M17 +++
M18 -
M19 -
M20 +
M21 ++
M22 +++
M23 -
M24 +++
M25 -
M26 -
M27 -
M28 +++
M29 +
M30 -
M31 +++
M32 -
M33 -
M34 +++
M35 -
M36 +
M37 ++
M38 -
M39 +++
M40 +++
M41 +++
M42 +++
M43 +++
M44 +++
M45 +++
M46 ++
M47 +++
M48 +++
M49 ++
M50 +
M51 +
M52 +
M53 +++
M54 +++
M55 ++
M56 +
M57 ++
M58 ++
M59 +++
M60 +++
C
MWSPEREAEAPAGGDPAGLLPPEWEEDEERMSFLFSAFKRSREVNSTDWDSKMGFWAPLVLS
HSRRQGVVRLRLRDLQEAFQRKGSVPLGLATVLQDLLRRGELQRESDFMASVDSSWISWGVG
VFLLKPLKWTLSNMLGDNKVPAEEVLVAVELLKEKAEEVYRLYQNSPLSSHPVVALSELSTL
CANSCPDERTFYLVLLQLQKEKRVTVLEQNGEKIVKFARGPRAKVSPVNDVD
D
*
ESDFMASVDSSWISWGVGVFLLKPLKWTLSNMLGDNKVPAEEV
WH-2
WH-1

Figure S3, related to Figure 2 : Sequence analysis and mapping of ER-localisation determinants of CHMP7^{NT}

A. Manual alignment of CHMP7^{NT} and VPS25. Secondary structural prediction of elements within CHMP7^{NT} using JPred [S2] and aligned against the secondary-structural elements obtained from the crystal structure of VPS25 (PDB 3CUQ chain C) [S3]. Residue numbering given for CHMP7. B. HeLa cells expressing the indicated GFP-CHMP7^{NT} proteins were imaged live. Mitotic cells were captured to maximise ER visibility. Scale bar is 10 μm. Images representative of all cells imaged and 3/3 captured images per mutation. Residues mutated in blocks of 4 sequential amino acids were are : M1, $W^2S^3P^4E^5$ – AAAA; M2, REAE - AAVA; M3, APAG - VAVA; M4, GDPA - AAAV; M5, GLLP – AAAA; M6, PEWE - AAAA; M7, EDEE - AAAA; M8, RMSF - AAAA; M9, LFSA - AAAV; M10, FKRS - AAAA; M11, REVN - AAAA; M12, STDW – AAAA; M13, DSKM – AAAA; M14, GFWA – AAAV; M15, PLVL – AAAA; M16, SHSR – AAAA; M17, RQGV – AAAA; M18, VRLR – AAAA; M19, LRDL – AAAA; M20, QEAF – AAVA; M21, QRKG – AAAA; M22, SVPL – AAAA; M23, GLAT – AAVA; M24, VLQD – AAAA; M25, LLRR-AAAA, M26, GELQ – AAAA; M27, RESD – AAAA; M28, FMAS – AAAA; M29, VDSS – AAAA; M30, WISW – AAAA; M31, GVGV – AAAA; M32, FLLK – AAAA; M33, PLKW – AAAA; M34, TLSN – AAAA; M35, MLGD – AAAA; M36, NKVP – AAAA; M37, AEEV – VAAA; M38, LVAV – AAVA; M39, ELLK – AAAA; M40, EKAE – AAAA; M41, EVYR – AAAA; M42, LYQN – AAAA; M43, SPLS – AAAA; M44, SHPV – AAAA; M45, VALS – AAAA; M46, ELST – AAAA; M47, LCAN – AAAA; M48, SCPD – AAAA; M49, ERTF – AAAA; M50, YLVL – AAAA; M51, LQLQ – AAAA; M52, KEKR – AAAA; M53, VTVL – AAAA; M54, EQ – AA; M55, NG – AA; M56, EKIV – AAAA; M57, KFAR – AAAA; M58, GPRA – AAAA; M59, KVSP – AAAA; M60, VNDV – AAAA. Images were scored for strength of ER localisation ((+++ normal), (++ reduced), (+ weak) or (- cytosolic). Colour-coded key to positions of residues on the homology model given for mutations that abolished ER-localisation. C. Sequence of CHMP7^{NT} with mutants (M18, M19, M23, M25, M26, M27, M30, M32, M33, M35, M38) that disrupt localisation to the ER highlighted in colours as per Figure S3B – mutations in the WH1 β2 - β3 insertion (underlined) are highlighted in red. D. Homology model of CHMP7^{NT} tandem WH-core (amino acids 19-224; δ107-148) based upon VPS25 3CUQ. Modelled structure in blue (WH1) and pink (WH2), position of mutants that disrupt localisation to the ER highlighted in colours as per Figure S3B. Position of insertion between β2 and β3 given as text sequence with δ118-128 region underlined and L127 highlighted. Residues highlighted in red correspond to amino acids that abolished ER localisation when mutated, as depicted in Figure S3B and S3C.

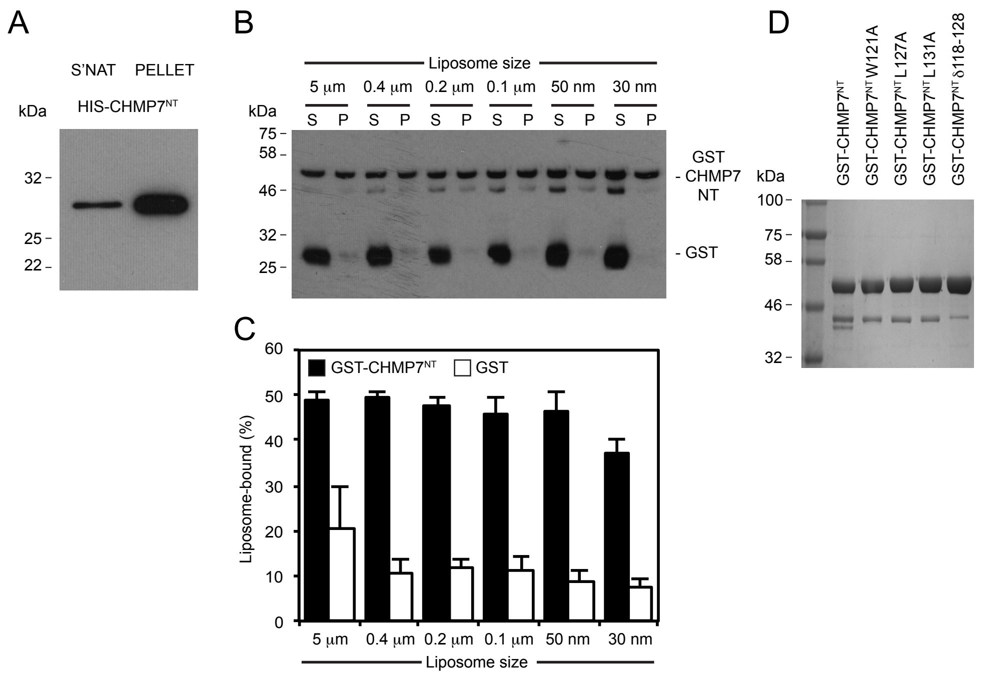
A
S'NAT
PELLET
kDa
HIS-CHMP7NT
32
25
22
B
Liposome size
5 µm
0.4 µm
0.2 µm
0.1 µm
50 nm
30 nm
kDa
S P S P S P S P S P S P
75
58
46
32
25
GST
- CHMP7
NT
- GST
C
GST-CHMP7NT
GST
Liposome-bound (%)
60
50
40
30
20
10
0
5 µm
0.4 µm
0.2 µm
0.1 µm
50 nm
30 nm
Liposome size
D
GST-CHMP7NT
GST-CHMP7NT W121A
GST-CHMP7NT L127A
GST-CHMP7NT L131A
GST-CHMP7NT δ118-128
kDa
100
75
58
46
32

Figure S4, related to Figure 3 : Characterisation of CHMP7 lipid binding

A. Resolved pellet and supernatant fractions of HIS-CHMP7^{NT} interaction with synthetic liposomes (60% DOPC, 20% DOPS, 20% DOPE) were analysed by western blotting with anti-HIS. Blot representative of 2 independent experiments, performed in duplicate and triplicate respectively. B. Resolved pellet and supernatant fractions of GST-CHMP7^{NT} incubated with synthetic liposomes (60% DOPC, 20% DOPS, 20% DOPE) of the indicated size. C. Quantification of binding from Figure S4B. Values quotes as mean ± S.E.M. from 3 independent experiments; two of these experiments were performed in duplicate and averaged. Statistical significance calculated using 1-way ANOVA with Tukey's multiple comparison test from experimental means. Significance was not achieved in any case. D. Coomassie stained gel of GST-CHMP7^{NT} mutations from Figure 3E.

Supplemental Experimental Procedures

Cell Culture

HeLa (a gift from Prof Martin-Serrano, KCL, and authenticated by STR profiling to ASN-002), GP2-293 (purchased from Clontech), CAL-51 (a breast carcinoma cell line with a normal diploid karyotype [S4], that were a gift from Prof Tutt, KCL, and authenticated by STR profiling to ANS-002) or Cos7 (a gift from Prof Ridley, KCL) cells were cultured in DMEM containing 10% FBS, Penicillin (100 U/ml) and Streptomycin (0.1 mg/ml). Stable cells lines were generated by transduction using MLV-based retroviruses as described previously [S5], and selected using Puromycin (200 ng/ml), G418 (500 μg/ml) or hygromycin (200 mg/ml) as necessary. Where necessary, cells were sorted to monoclonality by limiting dilution or FACS. Cell lines stably expressing Histone 2B-mCherry (H2B-mCh) and GFP-NLS have been described previously [S6].

Plasmids

The human CHMP7 coding sequence was amplified from Image Clone 5551762 (GE Healthcare) and cloned using in-frame 5' *EcoRI* and 3' *NotI* restriction sites into the retroviral packaging vectors pCMS28-GFP-*EcoRI-NotI-XhoI* (ENX; IRES-Puro), pNG72-mCh-ENX (IRES-Neo) or pNG72-HA-ENX (IRES-Neo) (MCS-modified versions of gifts from Dr Chad Swanson and Prof Mike Malim, KCL) [S7]. CHMP7^{NT} comprises residues 1-238. Deletions and mutations of CHMP7 were created by standard PCR-based molecular biology procedures and inserted into pCMS28-ENX, pCMS28-GFP-ENX or pNG72-HA-ENX similarly. All constructs were verified by sequencing. CHMP7 was rendered resistant to CHMP7-targeting siRNA oligo-1 through the introduction of silent mutations G217G, E218E, K219K, I220I, V221V, K222K using the following primer and its reverse complement : gcagaacggTgaAaaAatAgtAaaAtttgcccgagg. Altered bases generating silent mutations are indicated by capital type. Coding sequences were cloned *EcoRI-NotI* into pGEX or pET28a vectors for expression of recombinant proteins. To delete the loop between WH1 β2-β3, a Gly-Ser-Gly-Ser linker was inserted in place of residues 107-148 (CHMP7 δ107-148). To generate the chimaera of CHMP7^{NT-R}/CHMP4B, CHMP4B was first cloned into the *EcoRI* and *XhoI* sites of pNG72-HA-ENX. CHMP7^{NT-R} was then amplified *EcoRI-EcoRI* and cloned into the *EcoRI* sites of pNG72-HA-CHMP4B. CHMP4B was additionally cloned into the *EcoRI* and *XhoI* sites of pCMS28-GFP-ENX for creation of cell lines stably expressing GFP-CHMP4B. pX335-U6-Chimeric_BB-CBh-hSpCas9n(D10A) was a gift from Prof Zhang (Addgene plasmid # 42335).

For retroviral transduction, above constructs in retroviral packaging vectors were transfected with pVSVG into GP2-293 cells (Clontech). Supernatants were harvested, clarified by centrifugation (200 x g, 5 minutes), filtered (0.45 μm) and used to infect target cells in the presence of 8 μg/ml polybrene (Millipore) at MOI < 1. Antibiotic selection was applied after 48 hours.

CRISPR/Cas9 mediated editing

DNA encoding for monomeric NeonGreen (mNG [S8]) fluorescent protein was appended to the N-terminus of endogenous CHMP7 in CAL-51 cells (a near diploid breast carcinoma line) using a dual-nickase CRISPR approach to direct homology-repair (HR) mediated editing of the endogenous CHMP7 locus [S9]. mNG was selected based upon its reported 2.8-fold brightness when compared to GFP [S8]. Guide RNAs were selected using the guide RNA design tool maintained at http://crispr.mit.edu, using a 213nt stretch of CHMP7 genomic DNA centred around the start codon as the search template. Top-scoring upstream (<u>CCA</u>GGCTTGTGTTCGCAGCCTTG) and downstream guides were selected (1, TTCCG**ATG**TGGTCCCCGGAG<u>CGG</u>, offset of +12nt; 2, TCCG**ATG**TGGTCCCCGGAGC<u>GGG</u>, offset of +13nt; 3, TGGGGTTCCG**ATG**TGGTCCC<u>CGG</u>, offset of +7nt), synthesised and cloned into *BbsI* sites of pX335. The top 3 scoring pairs were trialled, although all produced edits to a similar degree of efficiency. Protospacer adjacent motifs (PAMs) are underlined, start codon is in bold and the indicated location of these guide RNAs is indicated on Figure S1C as daggers (upstream) and inverted daggers (downstream guides). A homology-repair (HR) template (Figure S1D)

comprising an N-terminal homology arm (800nt), a Kozak sequence, the coding sequence for mNG, an *EcoRI* linker followed directly by the start codon of CHMP7 and a C-terminal homology arm (930nt) was synthesized (Eurofins) and cloned into the promotorless vector pEX-K4 (Eurofins). In this template, the PAMs were mutated (silently, where necessary) to prevent nicking of the repaired genomic DNA by any residual Cas9 D10A in the edited cells. CAL-51 cells were seeded in 6-well plates and transiently transfected with 1000 ng of pEX-K4-HR-template (circular) and 500 ng of each of the pX335 pairs (upstream and downstream-1, upstream and downstream-2, upstream and downstream-3) using Lipofectamine 3000. Parallel transfections including pCR3.1-mCherry indicated that transfection efficiencies of approx. 40% were achieved. After 5 days, cells were analysed by FACS and the brightest mNG-positive cells (Figure S1F) were sorted singly into 96 well plates as we reasoned these would represent homozygous edits. After 2 weeks of growth, clones were analysed by PCR. Primer pairs internal to the HR-template (Figure S1E, primers 1 and 2) and primer pairs spanning the HR join (Figure S1E, primers 3 and 4) were designed. Pairs internal to the HR template were located in the 5' UTR and 1st exon of CHMP7 allowing discernment of the parental and edited genomes. Primer pairs spanning the HR join were located in the 5' UTR and the mNG insertion to allow us to determine mNG insertion at the correct locus. gDNA was obtained using QuickExtract (Epicentre; 25 μl of QuickExtract per well of the 96 well plate, 15 minutes 65 ^{o}C, 15 minutes at 68 ^{o}C and 10 minutes at 98 ^{o}C) 0.5 μl of gDNA product was combined in a standard 50 μl PCR containing the screening primers. Positive clones were retained and analysed by fluorescence microscopy and western blotting with CHMP7 antisera as described previously.

Antibodies

An antibody against GAPDH (MAB374) was from Millipore; Calnexin (ab22595) was from Abcam; Tubulin (DM1A) was from Sigma; CHMP2A (104771-AP) was from Proteintech; CHMP7 (16424-1-AP) was from Proteintech (note this antibody detected a CHMP7 siRNA-sensitive band at 50kDa and an siRNA insensitive band at 51kDa. Gels were run to maximally separate these bands). GFP (7.1/13.1) was from Roche; mCherry (ab167453) was from Abcam; HA (HA.11) was from Covance; RANBP3 (134052) was from Abcam; ERp57 (TO2) was from Sigma; EEA1 (C45B10) was from Cell Signaling Technology; GST (27457701V) was from GE Healthcare; HIS (2365) was from Cell Signaling Technology; Vinculin (V9131) was from Sigma; HSP90 (H114) was from Santa Cruz. Alexa conjugated secondary antibodies were from Invitrogen and HRP-conjugated secondary antibodies were from Millipore.

Subcellular Fractionation

Following the method of Graham, 2002 [S10], cells (approx. 100 million) were collected and swollen for 10 minutes in homogenisation buffer (0.25 M Sucrose, 1 mM EDTA, 10 mM Hepes (pH 7.4)) and broken by 10 passages through a 12 μm-spaced ball-bearing homogeniser (Isobiotec). Nuclei and cellular debris were pelleted by centrifugation (10 minutes at 1700 x g) and a post-nuclear supernatant was layered on top of a 13 ml continuous (0-25%) iodixanol gradient atop a 50% iodixanol cushion. The gradient was centrifuged at 150,000 x g for 15 hours using an SW28 Ti rotor (Beckmann) and 0.5 ml fractions were collected for analysis by SDS-PAGE and western blotting.

SDS-PAGE and western blotting

Cell lysates and fractions were denatured by boiling in Laemmli buffer and resolved using SDS-PAGE. Resolved proteins were transferred onto nitrocellulose by western blotting and were probed with the indicated antisera in 5% milk. HRP-conjugated secondary antibodies were incubated with ECL Prime enhanced chemiluminescent substrate (GE Healthcare) and visualized by exposure to autoradiography film.

Transient transfection of cDNA

HeLa and CAL-51 cells were transfected using Lipofectamine-3000 (Life Technologies) according to the manufacturers instructions. 293GP2 cells were transfected using linear 25-kDa polyethylenimine (PEI, Polysciences, Inc.)

siRNA transfections

HeLa cells were seeded at a density of 1E5 cells/ml and were transfected with siRNA at 20 nM, 2 hours after plating using RNAi-MAX (Invitrogen), for 72 hours. The following targeting sequences that have already been demonstrated to achieve potent and specific suppression of the targeted CHMP were employed: Control – Dharmacon Non-targeting control D-001810, CHMP7-1 (GGGAGAAGATTGTGAAGTTdTdT [S11], CHMP7-2 (GGAGGUGUAUCGUCUGUAUdTdT, M-015514-11). Given the similarity between the CHMP7 sequence targeted by oligo-1 and RANBP3, we ensured that oligos used in this study did not suppress endogenous RANBP3, whereas a RANBP3-targeting siRNA (Smartpool M-011484) effectively suppressed endogenous RANBP3 (Figure S1B).

Production of recombinant proteins

BL21 (DE3) * *E. coli*, expressing plasmids encoding GST- or HIS-tagged proteins, were resuspended in bacterial lysis buffer (20mM Hepes (pH 7.4), 500 mM NaCl, 3.5% glycerol and supplemented with Complete mini, EDTA-free protease inhibitor (Roche) and 1 mM PMSF. Cells were lysed by addition of lysosyme (1 mg/ml, 15 minutes), Triton X100 (0.25%, 15 minutes) and were snap frozen in liquid nitrogen. Cells were thawed on ice, clarified through addition of DNAse1 (20 μg/ml) and soluble proteins were collected by centrifugation at 28,000 x g for 30 minutes. Proteins were immobilised on Glutathione Sepharose 4b or Ni-NTA agarose resins, washed extensively in wash buffer (20mM Hepes, pH 7.4, 150mM NaCl, 3.5% Glycerol). Proteins were eluted from Glutathione Sepharose 4β resin in wash-buffer supplemented with 10mM reduced glutathione (pH 8) and were dialysed against wash-buffer. Eluted proteins were stored at -80 ^{o}C. HIS-tagged proteins expressed from pET28a were expressed and harvested similarly, barring that all buffers contained 20 mM imidazole, and that proteins were eluted with a step gradient of imidazole rather than glutathione.

Liposome binding assays

Liposome binding assays were performed as previously described [S12]. Briefly, Folch extract was resuspended at 10mg/ml in $CHCl_3$:MeOH (19:1) and was dried as a film onto a round-bottomed glass tube by overnight rotary evaporation. The lipid film was rehydrated at 10 mg/ml under rotation in sucrose buffer (200 mM Sucrose, 20 mM KCL, 20 mM Hepes, pH 7.4) for 1 hour. Alternatively, synthetic liposomes were prepared by drying mixtures of 1,2-dioleoyl-*sn*-glycero-3-phosphocholine (DOPC (60%)), 1,2-dioleoyl-*sn*-glycero-3-phosphoserine (DOPS (20%)) and 1,2-dioleoyl-*sn*-glycero-3-phosphoethanolamine (DOPE (20%)) and resuspending similarly. 2.5% 1-2-dioleoyl-*sn*-glycerol (DAG) or 1,2-dioleoyl-*sn*-glycero-3-phosphate (PA) was added if required. Synthetic lipids were from Avanti Polar Lipids. Insoluble matter was removed by centrifugation (1000 x g, 1 minute) and liposomes were generated by bath sonication (5 minutes). In Figure S4B, liposomes were generated by extrusion of the rehydrated synthetic lipids though indicated defined pore-size nitrocellulose filters (Whatmann) using an Avanti Mini-Extruder. Proteins were diluted to 7 μg/ml in osmotically-matched protein dilution buffer (20 mM Hepes, 120 mM NaCl, 1 mM EGTA, 0.2 mM $CaCl_2$, 1.5 mM $MgCl_2$, 1 mM DTT, 5mM KCl, pH 7.4, 1% BSA was added to enhance solubility) and were pre-cleared by ultracentrifugation at 120,000 x g for 45 minutes using a TLA100.3 rotor in a Beckmann Optima-MAX benchtop ultracentrifuge. 1 ml of protein mixture was then combined with 10 μl of liposomes and incubated with shaking at 30 ^{o}C. Liposomes were recovered by ultracentrifugation (120,000 x g for 30 minutes); supernatant and pellet fractions were resuspended in equal volumes of 1 x Laemmli buffer and analysed by western blotting. Band intensities were quantified by densitometry using ImageJ and liposome-bound fractions were calculated.

Fixed cell imaging

Cells were imaged using Nikon Eclipse microscopes teamed with confocal (CSU-X1 Andor Spinning Disc/Ixon3 EM-CCD) imaging systems. Images were processed in NIS Elements and exported to Photoshop for assembly into figures. HeLa cells were fixed in MeOH (for CHMP2A-staining) or 4% PFA and subject to processing for immunofluorescence as described previously [S13].

Live cell imaging

Cells stably expressing the indicated proteins, or edited to express fluorescent proteins, were plated in 4- or 8-chamber Stickyslides (Ibidi) adhered to a glass number 1 coverslip, or wells of a glass-bottomed 96 well plate (Ibidi). Cells were transfected with the indicated siRNA where necessary. For analysis of mNG- or GFP-CHMP7 recruitment, cells were transferred to a inverted spinning disc confocal microscope (Nikon Eclipse, teamed with CSU-X1 Andor Spinning Disc with Ixon3 EM-CCD) with attached environmental chamber and imaged live using a 100x oil-immersion objective, acquiring frames every 30 seconds. For imaging of mNG-CHMP7 edited CAL-51 cells, maximal laser power and 1 second exposures were needed to capture the weak signal, resulting in bleaching after approx. 20 frames. For imaging stable GFP-CHMP7 expressing cell lines, <300 ms exposures could be used. For enrichment of GFP-CHMP7 on the forming NE, background-corrected maximal fluorescence intensities on the telophase NE were normalised against those on regions of adjacent ER. In all cases, 405, 488 or 565 laser lines were used for illumination.

For analysis of nucleo-cytoplasmic compartmentalisation, as described in [S6], cells were synchronised using a double thymidine block and 54 hours after siRNA transfection (10.5 hours after release from the second thymidine block), cells were transferred to a inverted spinning disc confocal microscope with attached environmental chamber and imaged live for 4 hours using a 20x dry objective and a 1.5 x magnification lens, acquiring frames every 1-5 mins. The ratio of background-corrected, area-normalised, GFP-positive pixel intensities within the cytoplasm and mCh-H2B demarcated nuclei at the indicated intervals were obtained using NIS-elements. Typically 20 daughter cells per siRNA treatment were analysed and the indicated number of independent experiments were performed as described in the relevant figure legends.

Modelling

CHMP7 residues 1-238 comprising tandem WH domains identified by HHpred (with a 4 residue flexible linker replacing the insertion (residues 107-148)), was submitted to Swiss-Model server using a template-directed homology search, returning VPS25 (3CUQ). Models were built and exported from Swiss PDB-viewer.

Statistical analysis

2-tailed Student's T-tests, or ordinary 1-way ANOVA with the indicated post-hoc tests were used to assess significance between test samples and controls and were performed using GraphPad Prism. N-numbers given as the number of independent experiments, n-numbers given as the number of cells analysed.

Supplemental References

1. Bajorek, M., Schubert, H.L., McCullough, J., Langelier, C., Eckert, D.M., Stubblefield, W-M.B., Uter, N.T., Myszka, D.G., Hill, C.P., and Sundquist, W.I. (2009). Structural basis for ESCRT-III protein autoinhibition. Nat. Struct. Mol. Biol. *16*, 754–762.
2. Drozdetskiy, A., Cole, C., Procter, J., and Barton, G.J. (2015). JPred4: a protein secondary structure prediction server. Nucleic Acids Res. *43*, W389–W394.
3. Im, Y.J., and Hurley, J.H. (2008). Integrated structural model and membrane targeting mechanism of the human ESCRT-II complex. Dev. Cell *14*, 902–913.
4. Gioanni, J., Le François, D., Zanghellini, E., Mazeau, C., Ettore, F., Lambert, J.C., Schneider, M., and Dutrillaux, B. (1990). Establishment and characterisation of a new tumorigenic cell line with a normal karyotype derived from a human breast adenocarcinoma. Br. J. Cancer *62*, 8–13.
5. Carlton, J.G., Agromayor, M., and Martin-Serrano, J. (2008). Differential requirements for Alix and ESCRT-III in cytokinesis and HIV-1 release. Proc. Natl. Acad. Sci. U.S.A. *105*, 10541–10546.
6. Olmos, Y., Hodgson, L., Mantell, J., Verkade, P., and Carlton, J.G. (2015). ESCRT-III controls nuclear envelope reformation. Nature *522*, 236–239.
7. Gallois-Montbrun, S., Kramer, B., Swanson, C.M., Byers, H., Lynham, S., Ward, M., and Malim, M.H. (2007). Antiviral protein APOBEC3G localizes to ribonucleoprotein complexes found in P bodies and stress granules. J. Virol. *81*, 2165–2178.
8. Shaner, N.C., Lambert, G.G., Chammas, A., Ni, Y., Cranfill, P.J., Baird, M.A., Sell, B.R., Allen, J.R., Day, R.N., Israelsson, M., et al. (2013). A bright monomeric green fluorescent protein derived from Branchiostoma lanceolatum. Nat. Methods *10*, 407–409.
9. Cong, L., Ran, F.A., Cox, D., Lin, S., Barretto, R., Habib, N., Hsu, P.D., Wu, X., Jiang, W., Marraffini, L.A., et al. (2013). Multiplex genome engineering using CRISPR/Cas systems. Science *339*, 819–823.
10. Graham, J. (2002). Fractionation of Golgi, endoplasmic reticulum, and plasma membrane from cultured cells in a preformed continuous iodixanol gradient. Scientific World J. *2*, 1435-1439.
11. Morita, E., Sandrin, V., McCullough, J., Katsuyama, A., Hamilton, I.B., and Sundquist, W.I. (2011). ESCRT-III protein requirements for HIV-1 budding. Cell Host Microbe *9*, 235–242.
12. Cozier, G.E., Carlton, J., McGregor, A.H., Gleeson, P.A., Teasdale, R.D., Mellor, H., and Cullen, P.J. (2002). The phox homology (PX) domain-dependent, 3-phosphoinositide-mediated association of sorting nexin-1 with an early sorting endosomal compartment is required for its ability to regulate epidermal growth factor receptor degradation. J Biol Chem *277*, 48730–48736.
13. Carlton, J.G., Caballe, A., Agromayor, M., Kloc, M., and Martin-Serrano, J. (2012). ESCRT-III governs the Aurora B-mediated abscission checkpoint through CHMP4C. Science *336*, 220–225.
